# Trends in low birth weight across 36 states and union territories in India, 1993-2021

**DOI:** 10.1136/bmjgh-2024-016732

**Published:** 2025-06-16

**Authors:** Omar Karlsson, Akhil Kumar, Rockli Kim, SV Subramanian

**Affiliations:** 1 Duke University, Durham, North Carolina, USA; 2 Lund University, Lund, Sweden; 3 University of Toronto, Toronto, Ontario, Canada; 4 Division of Health Policy and Management, Korea University College of Health Science, Seoul, South Korea; 5 Interdisciplinary Program in Precision Public Health, Department of Public Health Sciences, Graduate School of Korea University, Seoul, South Korea; 6 Harvard Center for Population and Development Studies, Cambridge, Massachusetts, USA; 7 Department of Social and Behavioral Sciences, Harvard TH Chan School of Public Health, Boston, Massachusetts, USA

**Keywords:** India, Child health

## Abstract

**ABSTRACT:**

**Introduction:**

Low birth weight is an important measure of the health of pregnant women and newborns. We investigated the prevalence of low birth weights in India over nearly three decades to assess trends and convergence across states.

**Methods:**

Data came from five waves of the National Family Health Survey (1992–93 to 2019–21). The prevalence of low birth weight was estimated. To explore the sensitivity of our results to missing birth weight data—since the completeness of birth weight information has changed drastically—we also estimated prevalence from multiple imputation models, Heckman selection models, and by reweighting the data so that socioeconomic characteristics of children with birth weight data matched across surveys.

**Results:**

The overall prevalence of low birth weight in India declined from 26% to 18% during the period. The 2019–21 survey revealed that four states, Uttar Pradesh, Bihar, Maharashtra, and West Bengal accounted for almost half of all low-birth-weight births in India. The Pearson’s correlation between the prevalence of low birth weight in 1992–93 and percentage point change across the period was −0.85, suggesting convergence between states, where states with greater prevalence in 1992–93 had faster declines. Convergence was robust across sensitivity specifications.

**Conclusion:**

State-level convergence indicates a potential 'catch-up' phenomenon, where states with initially higher prevalence have experienced greater declines. This finding suggested a possible impact of interventions prompted by dire figures in the earliest surveys, yet also stresses the necessity for continued interventions across all states to maintain and further progress. Our analysis, however, warrants a cautious interpretation due to data limitations. However, we observed convergence in the prevalence of low birth weight across states in all sensitivity specifications.

**WHAT IS ALREADY KNOWN ON THIS TOPIC**

**WHAT THIS STUDY ADDS**

- This study provides a comprehensive analysis of trends in low birth weight across Indian states from 1993 to 2021, utilising data from five waves of the National Family Health Survey. It highlights not only the overall decline in prevalence of low birth weight but also the state-level convergence in these rates.

**HOW THIS STUDY MIGHT AFFECT RESEARCH, PRACTICE OR POLICY**

- Despite the overall progress, the persistence of high prevalence of low birth weight in certain states highlights the need for ongoing efforts to address maternal and neonatal health disparities. Despite improvements, data collection at healthcare facilities must also be further enhanced, to provide quality data for decision making across India.

## Introduction

 Birth weight is a widely used indicator of newborn health and is a predictor of future childhood and adult health.^1 2^ Low birth weight often signals prenatal adversities inhibiting optimal growth — such as underlying maternal health issues and poor nutrition — which is also detrimental to cognitive development and susceptibility to chronic conditions in later life.^3 4^ Levels and trends in birth weight thus have profound implications for long-term population health trajectories. Weighing newborns is a cornerstone of neonatal health assessment, providing timely insight into potential risks and directing subsequent medical interventions.^5^,^8^

In a country as diverse as India, state-level considerations are important. Each state has unique culture, governance, and socio-economic dynamics. While some states have witnessed rapid progress, others have faced greater challenges. Considering the under-five mortality rate per 1000 births: the best performing states, Puducherry and Kerala had a rate of 4 and 5 — a level observed in high income countries such as the United States and Canada — while the worst performing states, Uttar Pradesh and Bihar, had 56 and 60 under-five deaths per 1000 births — a level observed in low-income countries such as Afghanistan and Zambia.^9 10^ Recognising these disparities, India’s health policy has often been decentralised, enabling states to tailor interventions.

Yet, in the midst of persistent disparities, a trend of convergence in several child health outcomes across states has been observed.^11^ This trend offers hope but also raises new questions: Is birth weight, a crucial determinant of newborn and child health, also showing convergence? Our study examines changes in the prevalence of low birth weight across Indian states from 1993 to 2021. By doing so, it informs the discourse on neonatal health in India, intra-national disparities in health outcomes, and convergence across states over time.

### Data

This study utilised data from the National Family Health Survey (NFHS), a part of the international Demographic and Health Surveys program.^12^ Specifically, we considered data from five waves of the NFHS — conducted in 1992–93, 1998–99, 2005–06, 2015–16, and 2019–21.^13^,^17^ The NFHS is a nationally representative survey collecting extensive data on a variety of health and demographic indicators. Each wave of the NFHS employs a multi-stage sampling methodology that ensures representation across India’s geography and socioeconomic strata.^18^ In each household, all women 15–49 were interviewed in NFHS-3–5, ever-married women aged 13–49 in NFHS-1, and ever-married women aged 15–49 in NFHS-2. Data were collected on their health and their children’s health. Information on household characteristics was collected in a household questionnaire. Response rates exceeded 90% in all five waves.^13^,^17^

We considered live births occurring within 5 years of each interview. However, in NFHS-1, birth weight information was only recorded for children under 4 years old and in NFHS-2 for children under 3 years old. The total sample size aggregated across the five waves was 626 087. After excluding cases with missing birth weight data our final analytical sample consisted of over 440 200 live births (table 1).

**Table 1:** Study sample size from the five National Family Health Surveys (NFHS), 1993–2021

		Low birth weight			Small size at birth
Survey round (year)	Full sample	Not weighed	Missing	Included	Included
NFHS-1 (1992–93)	48 959	37 728 (77.1%)	3239 (6.6%)	7992 (16.3%)	48 285 (98.6%)
NFHS-2 (1998–99)	33 026	22 756 (68.9%)	1619 (4.9%)	8651 (26.2%)	32 909 (99.6%)
NFHS-3 (2005–06)	51 555	27 780 (53.9%)	2829 (5.5%)	20 946 (40.6%)	50 646 (98.2%)
NFHS-4 (2015–16)	259 627	57 451 (22.1%)	8831 (3.4%)	193 345 (74.5%)	253 213 (97.5%)
NFHS-5 (2019–21)	232 920	19 358 (8.3%)	4296 (1.8%)	209 266 (89.8%)	229 375 (98.5%)
All waves	626 087	165 073 (26.4%)	20 814 (3.3%)	440 200 (70.3%)	614 428 (98.1%)

NFHS, National Family Health Survey.

### Outcomes

The primary outcome variable for this analysis was low birth weight, defined as birth weight below 2500 grams. Given the logistical challenges and the lack of widespread hospital deliveries, especially in the earlier years covered by this study, it is noteworthy that birth weights reported in the NFHS derive from two sources: (1) from health cards where available; and (2) from maternal recall in instances where health cards were absent. Further, many children were not weighed, especially in the earlier surveys: therefore, we also show our results using the mother’s subjective assessment of the size of her child at birth, specifically whether the child was very small or smaller than average (online supplemental table 1).

We present 95% confidence intervals (CIs) based on standard errors adjusted for clustering at the level of primary sampling units. CIs for prevalence measure were logit adjusted to ensure that they remained between 0 and 100 (after scaling by 100 to obtain percentage prevalence). Further, for estimated changes in prevalence across times, we present p-values.

### Harmonising state boundaries across surveys

Currently, India comprises 28 states and eight union territories. However, the configuration of states and union territories has changed over time. For example, in 1993, there were 25 states and seven union territories. This redefinition of state boundaries complicates the process of creating repeated cross-sections of states and union territories that are consistent over the period. A common method to address this is to merge recent state configurations to match older ones. For instance, data from 2021 for Uttarakhand and Uttar Pradesh might be combined to represent them as one state, as they were once combined as Uttar Pradesh in the 1990s. Yet, representing current data in old state boundaries does not provide accurate contemporary insights, making it less relevant for present day state level policy discussions. We addressed this challenge by mapping districts from older surveys to their current state configurations based on data from the Survey of India (see Subramanian *et al,*^19^ for details). Specifically, for the 1993 and 1999 surveys, districts were reassigned to their current state when relevant. District data were unavailable for the 2006 surveys, so values for Andhra Pradesh were assigned to Telangana and values for Jammu & Kashmir were assigned to Ladakh. In the 2016 survey, Kargil and Leh districts (in Jammu & Kashmir) were assigned to Ladakh: a new union territory. Dadra & Nagar Haveli and Daman & Diu were merged into one union territory. No changes were made for the 2021 survey.

### Supplementary and sensitivity analyses

The percentage of children who were weighed at birth changed vastly over the period and was further patterned by socioeconomic characteristics: Those in the poorest households and born to women without education were far less likely to be weighed at birth (online supplemental tables 2, 3). Our sensitivity analyses aim to account for missing birth weight data based on socioeconomic and other characteristics to improve the representativeness of the prevalence measure in the earlier surveys. We assessed the sensitivity of our results to changes in the prevalence of weighing over time using three different approaches.

**Figure 1:**
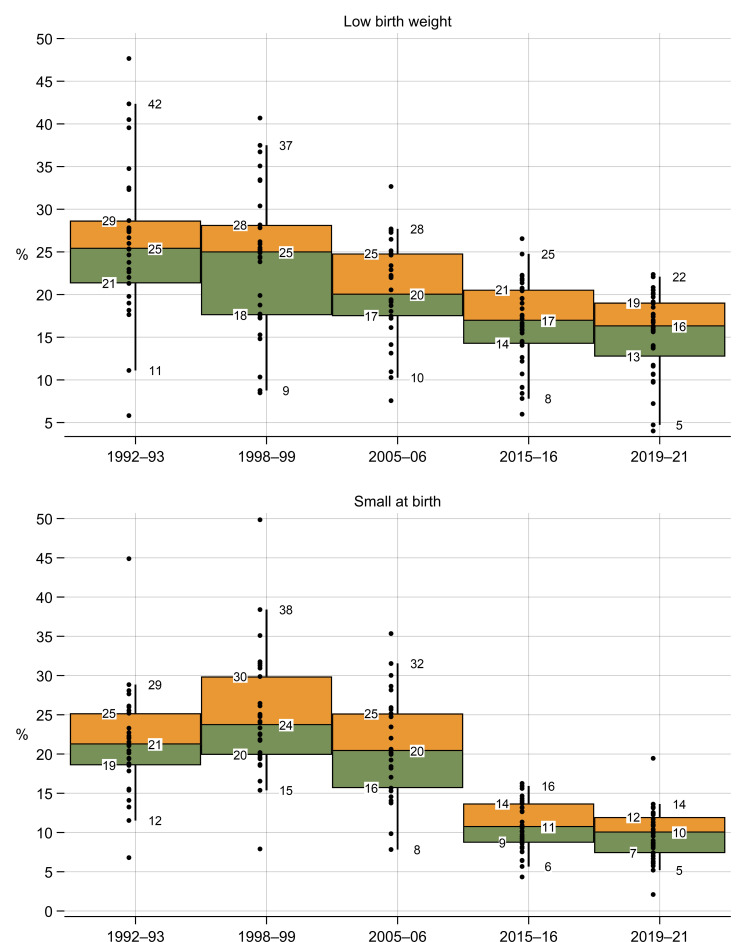
Summary distribution of state and union territory-level prevalence, 1993–2021: Low birth weight and small size at birth. Notes: Percentiles 5 and 95 (line) and 25, 50, and 75 (box) are shown. Dots indicate state estimates. States were equally weighted for the median and percentiles.

**Figure 2:**
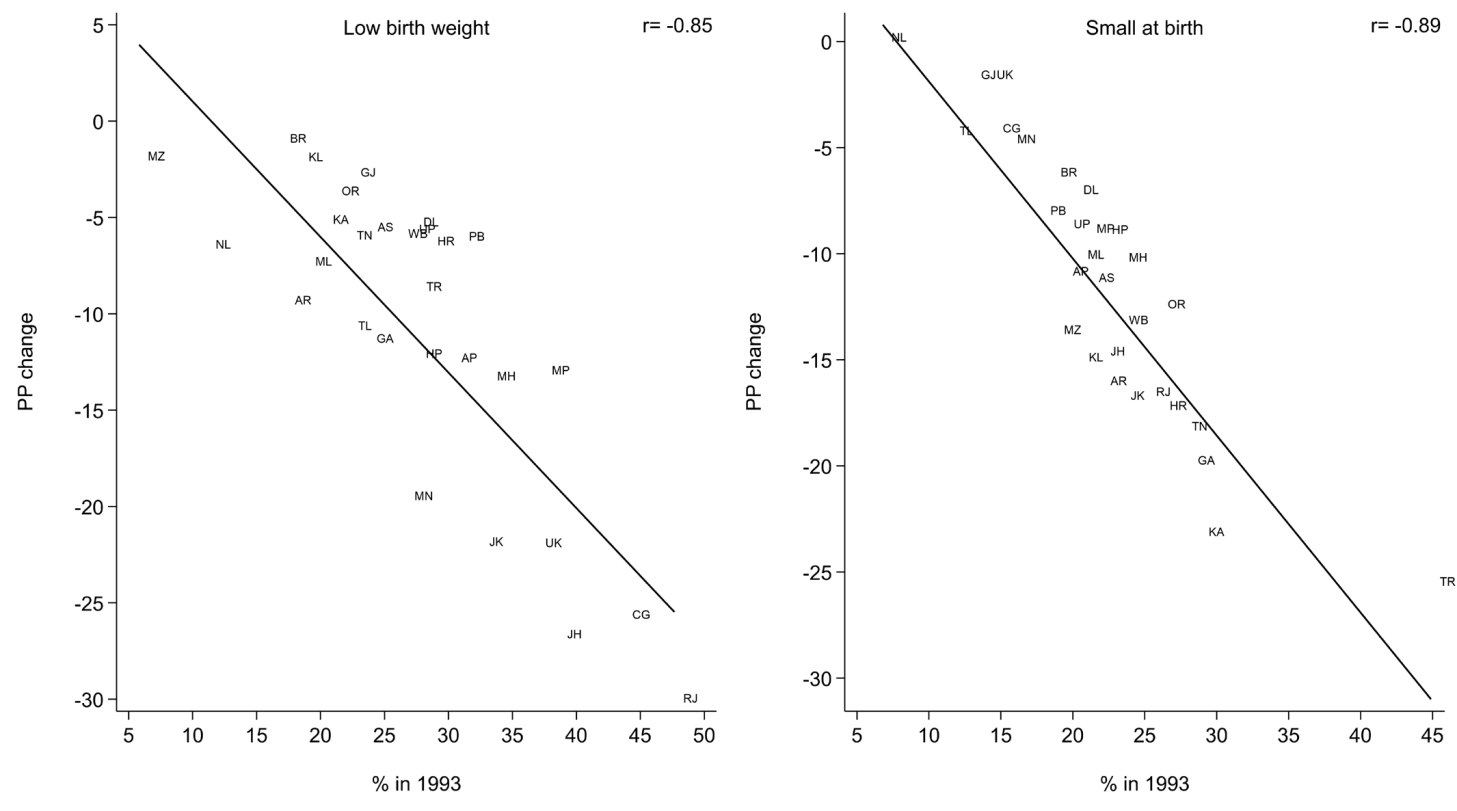
Relationship between prevalence (%) in 1993 and percentage points (pp) change for 1993–2021: Low birth weight and small size at birth Notes: State labels were randomly displaced by at most 10% on either axis for readability. Andhra Pradesh (AP), Arunachal Pradesh (AR), Assam (AS), Bihar (BR), Chhattisgarh (CG), Goa (GA), Gujarat (GJ), Haryana (HR), Himachal Pradesh (HP), Jammu & Kashmir (JK), Jharkhand (JH), Karnataka (KA), Kerala (KL), Madhya Pradesh (MP), Maharashtra (MH), Manipur (MN), Meghalaya (ML), Mizoram (MZ), Nagaland (NL), NCT of Delhi (DL), Odisha (OR), Punjab (PB), Rajasthan (RJ), Tamil Nadu (TN), Telangana (TL), Tripura (TR), Uttar Pradesh (UP), Uttarakhand (UK), West Bengal (WB).

First, we imputed the missing birth weight using multiple imputations, for each survey separately, where missing birth weight was imputed using sex, mother’s level of education (none, primary, secondary, higher), a wealth index^20^ (converted into survey specific z-score and specified using linear and squared terms), place of birth (outside health facility, public health facility, private health facility, other type of health facility), and size at birth (very small, below average, average, above average, very large, unknown to mother) as independent variables (online supplemental tables 4, 5 and figures 1, 2). We also included the average of these variables within primary sampling units as independent variables. These multiple imputations predict the expected birth weight for children with missing birth weight, based on the covariates, which was then used to calculate prevelance of low birth weight. Note, however, that multiple imputations will not fully eliminate bias when missingness is related to birth weight in ways other than captured by the covariates.

Second, we accounted for missing birth weight data using Heckman selection models (online supplemental table 6, 7 and figures 3, 4). Selection into the main analysis (ie, whether the child was weighed or not) was first modelled for each survey separately using sex, mother’s level of education, wealth index z-score, place of birth, and a dummy coded variable for state. All covariates except place of birth were also included in the second stage regression, from which prevalence of low birth weight was estimated (holding other covariates at their state-specific means). We also report results for whether a child was weighed at birth.

Third, we reweighted the analysis samples in waves 1–4 to match the distribution of socioeconomic strata in wave 5 to improve the representativeness of the earlier samples (online supplemental tables 8, 9 and figures 5, 6). The strata were a unique combination of household wealth index deciles (survey specific), state, and sex. Since characteristics such as the mother’s level of education and health facility deliveries have vastly improved across surveys, we do not include these as strata. In effect, the reweighting constructs a sample of births that have the same characteristics in all surveys. Note, however, that some strata from the earliest surveys may have had few or no observations.

We also provide estimates of the number of low birth weight births for the 2021 survey: estimated using the prevalence of low birth weight and estimated number of births, overall and by state (online supplemental table 10 and figure 7). The estimated number of births for each state were estimated by multiplying the proportion of births occurring in each state observed in the NFHS 2021 data by the total number of births for India in 2020 from the United Nations World Population Prospects (which was just over 23 million).^21^

Finally, we show socioeconomic and demographic patterns in low birth weight (online supplemental table 11).

### Patient and public involvement

Patients were not involved in this study.

### Compliance with ethical standards

This project used publicly accessible secondary data obtained from the Demographic and Health Survey website. These activities did not meet the regulatory definition of human subject research according to The Harvard Longwood Campus Institutional Review Board Decision Tool.

## Results

The nationwide full sample increased from 48 959 in 1993 to 232 920 in 2021 (table 1). Further, the proportion of these births that were weighed increased over time, resulting in 7 992 births being included in the analysis in 1993 (16% of the full sample) to 209 266 in 2021 (90% of the full sample).

There was a noticeable decrease in the state-level median prevalence of low birth weight across surveys (figure 1): from 25% in 1993 (interquartile range (IQR) 21 to 29) and 1999 (IQR 18 to 28) to 20% (IQR 17 to 25) in 2006; 17% (IQR 14 to 21) in 2016; and finally, 16% (IQR: 13 to 19) in 2021.

Similarly, the overall India prevalence of low birth weight declined from 26% in 1993 to 18% in 2021 (table 2). The greatest prevalence of low birth weight in 1993 was observed in Rajasthan (48%) and Chhattisgarh (42%) while the lowest prevalence was observed in Mizoram (6%) and Nagaland (11%). In 2021, the greatest prevalence was observed in Punjab (22%) and NCT of Delhi (22%) while the lowest prevalence was still observed in Mizoram (4%), Nagaland (5%), Manipur (7%).

**Table 2:** Prevalence (%) by survey: Low birth weight and small size at birth

	Low birth weight					Small at birth				
	1992–93	1998–99	2005–06	2015–16	2019–21	1992–93	1998–99	2005–06	2015–16	2019–21
India	26 (24, 27)	23 (21, 24)	21.5 (20.7, 22.4)	18.2 (17.9, 18.5)	18.2 (18.0, 18.5)	21.5 (20.8, 22.1)	24.5 (23.8, 25.3)	21 (20, 22)	12.3 (12.1, 12.6)	10.8 (10.5, 11.0)
States:										
Andhra Pradesh	28	18	19	18	16	19	15	8	9	9
	(20, 37)	(13, 24)	(16, 23)	(16, 19)	(15, 18)	(17, 22)	(13, 19)	(6, 10)	(7, 10)	(7, 10)
Arunachal Pradesh	20	25	14	11	11	22	26	29	13	6
	(13, 29)	(18, 34)	(10, 19)	(9, 12)	(9, 12)	(19, 27)	(22, 32)	(24, 34)	(12, 15)	(5, 7)
Assam	22	33	19	16	16	21	22	20	16	10
	(15, 31)	(26, 42)	(15, 24)	(15, 17)	(15, 17)	(19, 24)	(19, 25)	(18, 23)	(15, 18)	(9, 11)
Bihar	18	15	28	14.4	17	19	19	19	14	12.4
	(12, 25)	(10, 23)	(22, 34)	(13.7, 15.1)	(16, 18)	(17, 21)	(18, 21)	(16, 22)	(13, 15)	(11.6, 13.3)
Chhattisgarh	42	28	17	13	16	15	25	15	10	11
	(27, 60)	(16, 45)	(13, 22)	(12, 14)	(15, 17)	(12, 20)	(20, 31)	(13, 18)	(9, 11)	(10, 13)
Goa	25	25	22	22	14	28	32	20	9	8
	(21, 30)	(19, 32)	(19, 26)	(18, 27)	(10, 19)	(25, 31)	(25, 38)	(17, 23)	(7, 13)	(6, 12)
Gujarat	21	20	22	19	19	13	25	22	14	12
	(18, 25)	(16, 24)	(19, 25)	(18, 20)	(17, 20)	(12, 15)	(22, 28)	(19, 25)	(12, 15)	(11, 13)
Haryana	26	24	33	20	21	26	19	18	9	9
	(18, 36)	(19, 31)	(28, 38)	(19, 22)	(19. 22)	(24, 29)	(16, 21)	(15, 22)	(8, 11)	(8, 10)
Himachal Pradesh	28	35	25	20	16	22	24	20	14	13
	(21, 36)	(29, 42)	(21, 29)	(18, 22)	(14, 18)	(20, 25)	(20, 28)	(17, 23)	(12, 16)	(11, 15)
Jharkhand	41	37	19	14.5	16	22	20	23	10	7.4
	(32, 49)	(24, 53)	(15, 24)	(13.6, 15.5)	(15, 17)	(18, 27)	(16, 24)	(20, 27)	(9, 11)	(6.6, 8.3)
Karnataka	21	17	19	17	16	29	31	23	9	6
	(17, 27)	(14, 22)	(17, 21)	(16, 19)	(15, 17)	(26, 31)	(28, 34)	(21, 26)	(7, 10)	(5, 7)
Kerala	18	18	16	15	16	22	25	14	8	7
	(16, 21)	(15, 21)	(13, 20)	(14, 17)	(15, 18)	(20, 24)	(22, 28)	(12, 17)	(6, 9)	(6, 8)
Madhya Pradesh	35	33	23	22	20.5	21	38	25	13	12
	(29, 41)	(26, 41)	(20, 27)	(21, 23)	(19.7, 21.4)	(19, 23)	(36, 41)	(22, 28)	(12, 14)	(11, 13)
Maharashtra	32	26	22	20	20	23	24	18	11	13
	(28, 36)	(23, 30)	(20, 25)	(18, 21)	(19, 21)	(21, 26)	(22, 27)	(16, 21)	(10, 12)	(12, 15)
Manipur	27	10	13	9	7	16	20	17	14	11
	(18, 37)	(7, 15)	(11, 16)	(8, 10)	(6, 9)	(13, 19)	(17, 24)	(15, 20)	(13, 15)	(9, 13)
Meghalaya	19	15	18	12	12	20	31	26	9	10
	(14, 26)	(10, 22)	(14, 23)	(11, 14)	(10, 13)	(16, 25)	(27, 36)	(21, 31)	(8, 11)	(9, 12)
Mizoram	6	9	8	6	4	19	26	15	8	5
	(3, 10)	(6, 13)	(6, 10)	(5, 8)	(3, 5)	(15, 23)	(21, 32)	(12, 17)	(7, 10)	(4, 6)
Nagaland	11	8	11	8	5	7	20	16	11	7
	(1, 53)	(2, 30)	(7, 16)	(6, 10)	(4, 6)	(5, 10)	(16, 25)	(14, 18)	(10, 13)	(6, 8)
Odisha	23	25	21	21	19	26	22	25	14	14
	(16, 32)	(19, 32)	(17, 24)	(20, 22)	(18, 20)	(24, 28)	(19, 25)	(22, 28)	(13, 15)	(13, 15)
Punjab	29	24	28	17	22	18	19	30	13	10
	(21, 38)	(18, 31)	(23, 32)	(16, 19)	(21, 24)	(16, 20)	(16, 22)	(27, 34)	(11, 14)	(9, 11)
Rajasthan	48	30	27	21	18	25	35	26	10.6	8.7
	(36, 59)	(25, 36)	(22, 33)	(20, 22)	(17, 19)	(23, 27)	(33, 37)	(23, 29)	(9.9, 11.4)	(8.0, 9.4)
Sikkim		24	10	8	10		20	14	4	10
		(17, 33)	(8, 14)	(7, 11)	(6, 16)		(16, 24)	(11, 18)	(3, 6)	(7, 15)
Tamil Nadu	23	17	17	16	17	28	32	28	10	10
	(19, 27)	(15, 20)	(15, 20)	(15, 17)	(16, 18)	(25, 30)	(29, 35)	(25, 31)	(9, 11)	(9, 11)
Telangana	24	19	19	16	14	12	8	8	8	7
	(17, 32)	(15, 24)	(16, 23)	(14, 18)	(13, 15)	(9, 14)	(5, 12)	(6, 10)	(6, 9)	(6, 8)
Tripura	28	25	27	17	20	45	50	35	14	19
	(20, 36)	(16, 36)	(22, 33)	(15, 20)	(18, 22)	(40, 50)	(43, 56)	(30, 41)	(12, 17)	(17, 22)
Uttar Pradesh	25	37	25	20.7	20.2	19	22	21	15.6	11
	(18, 35)	(29, 45)	(21, 29)	(20.0, 21.4)	(19.5, 20.8)	(18, 21)	(21, 24)	(19, 22)	(15.0, 16.2)	(10, 12)
Uttarakhand	40	41	25	25	18	14	23	21	15	13
	(28, 53)	(26, 58)	(20, 30)	(22, 27)	(16, 20)	(11, 18)	(17, 31)	(17, 24)	(13, 16)	(11, 15)
West Bengal	25	25	23	17	19	26	23	25	13	12
	(21, 29)	(21, 30)	(20, 26)	(15, 18)	(18, 20)	(23, 28)	(19, 26)	(22, 28)	(11, 14)	(11, 14)
Union territories:										
Andaman & Nico- bar Islands				16	17				6	11
				(12, 21)	(13, 22)				(3, 9)	(8, 15)
Chandigarh				22	17				11	8
				(16, 30)	(11, 24)				(7, 17)	(5, 14)
Dadra & Nagar Haveli				22	21				6	11
& Daman & Diu				(18, 27)	(17, 25)				(4, 10)	(8, 16)
Jammu & Kashmir	33	28	19	14	11	23	30	32	11	6
	(24, 43)	(20, 38)	(14, 26)	(13, 15)	(10, 12)	(21, 25)	(26, 34)	(28, 35)	(10, 12)	(5, 7)
Ladakh				9	12				8	6
				(6, 13)	(8, 16)				(6, 12)	(4, 9)
Lakshadweep				18	10				16	2
				(13 26)	(6, 14)				(11, 23)	(0.9, 5)
Nct of Delhi	27	26	26	27	22	20	17	10	9	13
	(24, 31)	(22, 31)	(22, 31)	(22, 32)	(20, 24)	(18, 22)	(14, 20)	(8, 13)	(7, 12)	(12, 15)
Puducherry				16	14				6	8
				(13, 19)	(10, 19)				(4, 9)	(5, 13)

Notes: 95% confidence intervals are shown below estimates. The colours represent quintiles. Estimates are rounded to whole numbers (except when confidence intervals would overlap).

Low birth weight quintiles:
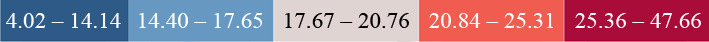

Small at birth quintiles:
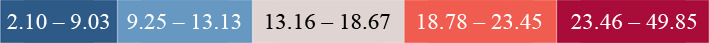

In terms of smaller than average size at birth (subjectively assessed by the mother) in 1993, the greatest prevalence was observed in Tripura (45%) and Karnataka (29%) and the lowest prevalence was observed in Nagaland (7%) and Telangana (12%). In 2021, the greatest prevalence of small birth size as assessed by the mother was also observed in Tripura (19%) and Odisha (14%), while the lowest prevalence was observed in Lakshadweep (2%) and Mizoram (5%).

Overall, there was an 8 percentage point (pp) decrease in the prevalence of low birth weight and 11 pp decrease in the prevalence of small birth size between the 1993 and 2021 surveys (table 3). The greatest decline in low birth weight was observed in Rajasthan (30 pp) and Chhattisgarh (26 pp) while the smallest decline was observed in Bihar (1 pp), Kerala (2 pp), and Mizoram (2 pp). For small birth size as assessed by the mother, the largest decline was observed in Tripura (25 pp) and Karnataka (23 pp) while the smallest decline was in Nagaland (0 pp), Uttarakhand (2 pp), and Gujarat (2 pp).

**Table 3:** Percentage point (pp) change in prevalence across surveys: Low birth weight and small size at birth

	Low birth weight					Small at birth				
	1992/3–	1992/3–	1998/9–	2005/6–	2015/6–	1992/3–	1992/3–	1998/9–	2005/6–	2015/6–
	2019/21	1998/9	2005/6	2015/6	2019/21	2019/21	1998/9	2005/6	2015/6	2019/21
India	−8***	−3***	-1	−3***	0	−10.7***	3***	−3***	−9***	−1.6***
	(−9, –6)	(−5,–1)	(−3, 0.4)	(−4,–2)	(−0.4, 0.4)	(-11.4,–10.0)	(2, 4)	(−4, –2)	(−10, –8)	(−1.9, –1.2)
States:										
Andhra Pradesh	−12***	−10*	2	-2	-1	−11***	−4**	−8***	0.9	−0.2
	(−20, –3)	(−20, 0.2)	(−5, 8)	(−6, 2)	(−4, 0.9)	(−14, –8)	(−8, –0.1)	(−11, –4)	(−1, 3)	(−2, 2)
Arunachal Pradesh	−9**	5	−11**	-3	−0.1	−16***	4	2	−15***	−7***
	(−18, –0.8)	(−6, 17)	(−20, –2)	(−8, 1)	(−2, 2)	(−20, –12)	(−2, 10)	(−5, 9)	(−21, –10)	(−9, –5)
Assam	-6	11*	−14***	-4	0.4	−11***	0.5	-2	−4**	−6***
	(−14, 2)	(−0.0, 23)	(−24, –4)	(−8, 1)	(−1, 2)	(−14, –8)	(−4, 5)	(−6, 3)	(−7, –0.9)	(−8, –5)
Bihar	−0.9	-2	12***	−13***	2***	−6***	0.8	−0.2	−5***	−2***
	(−8, 6)	(−12, 7)	(3, 22)	(−19, –7)	(1, 3)	(−8, –4)	(−2, 4)	(−4, 3)	(−8, –2)	(−3, –0.5)
Chhattisgarh	−26***	−15	−10	−5**	3***	−4*	9***	−10***	−5***	1
	(−44, –9)	(−37, 8)	(−26, 5)	(−9, –0.3)	(2, 5)	(−8, 0.0)	(3, 16)	(−15, –4)	(−7, –3)	(−0.6, 3)
Goa	−11***	−0.1	-3	0	−8***	−20***	3	−12***	−10***	-1
	(−17, –6)	(−8, 8)	(−10, 4)	(−6, 6)	(−14, –2)	(−24, –15)	(−4, 11)	(−19, –4)	(−15, –6)	(−6, 3)
Gujarat	-3	-1	2	−3*	−0.5	-2	12***	-3	−8***	−2**
	(−6, 0.8)	(−7, 4)	(−3, 7)	(−6, 0.5)	(−2, 1)	(−4, 0.5)	(8, 15)	(−7, 1)	(−11, –5)	(−4, –0.2)
Haryana	-5	-2	8**	−12***	0.1	−17***	−8***	−0.4	−9***	−0.5
	(−14, 3)	(−12, 9)	(0.4, 16)	(−17, –7)	(−2, 2)	(−20, –14)	(−11, –4)	(−5, 4)	(−12, –5)	(−2, 1.0)
Himachal Pradesh	−12***	8	−10**	−5**	−4**	−9***	2	-4	−6***	−0.5
	(−20, –4)	(−3, 18)	(−18, –2)	(−10, –0.6)	(−7, –0.9)	(−12, –5)	(−3, 7)	(−9, 1)	(−10, –3)	(−3, 2)
Jharkhand	−25***	-3	−18**	−5*	1*	−15***	-2	4	−14***	−2***
	(−34, –16)	(−20, 14)	(−34, –3)	(−9, 0.1)	(−0.2, 2)	(−19, –10)	(−8, 4)	(−2, 9)	(−17, –10)	(−3, –1)
Karnataka	−5**	-4	1	-2	-1	−23***	2	−8***	−15***	−3***
	(−11, –0.4)	(−10, 2)	(−3, 6)	(−4, 1)	(−3, 0.6)	(−26, –20)	(−1, 6)	(−12, –4)	(−18, –12)	(−4, –1)
Kerala	-2	−0.6	-1	−0.6	0.8	−15***	4*	−11***	−6***	−0.9
	(−5, 1)	(−4, 3)	(−6, 3)	(−4, 3)	(−2, 3)	(−17, –13)	(−0.1, 7)	(−15, –7)	(−9, –4)	(−3, 0.8)
Madhya Pradesh	−14***	-1	−10**	-2	−1**	−9***	17***	−14***	−12***	−0.9
	(−21, –8)	(−11, 8)	(−18, –2)	(−5, 2)	(−2, –0.2)	(−11, –6)	(14 21^)^	(−17, –10)	(−14, –9)	(−2, 0.2)
Maharashtra	−12***	−6**	−4*	−3*	0.5	−10***	0.9	−6***	−8***	2**
	(−16, –8)	(−12, –1)	(−8, 0.5)	(−5, 0.2)	(−1, 2)	(−13, –7)	(−3, 4)	(−10, –2)	(−11, –5)	(0.5, 4)
Manipur	−19***	−16***	3	−4***	−2**	−5**	4*	-3	−3**	−3**
	(−29, –10)	(−27, –6)	(−2, 8)	(−7, –1)	(−4, –0.2)	(−8, –0.9)	(−0.3, 9)	(−7, 1)	(−6, –0.7)	(−5, –0.5)
Meghalaya	−7**	-4	3	−6**	−0.5	−10***	11***	-5	−17***	1
	(−14, –1)	(−13, 4)	(−4, 11)	(−10, –1)	(−3, 2)	(−15, –5)	(4 17^)^	(−12, 2)	(−22, –11)	(−0.6, 3)
Mizoram	-2	3	-1	-2	−2**	−14***	7**	−12***	−6***	−3***
	(−5, 2)	(−2, 7)	(−5, 3)	(−4, 1.0)	(−4, –0.1)	(−1, –9)	(0.8, 14)	(−17, –6)	(−9, –4)	(−5, –1)
Nagaland	-6	-3	2	-3	−3***	0.2	13***	−5*	−4***	−4***
	(−28, 15)	(−27, 22)	(−10, 15)	(−8, 1)	(−5, –0.9)	(−2, 3)	(9 18^)^	(−9, 0.2)	(−7, –2)	(−6, –2)
Odisha	-4	2	-4	0.2	−2**	−12***	−4**	3*	−11***	−0.1
	(−12, 4)	(−8, 12)	(−12, 3)	(−3, 4)	(−3, –0.1)	(−15, –10)	(−8, –0.6)	(−0.6, 7)	(−14, –8)	(−2, 1)
Punjab	-6	-5	4	−11***	5***	−8***	0.8	11***	−17***	−3***
	(−15, 3)	(−16, 6)	(−4, 12)	(−15, –6)	(3 7)	(−10, –5)	(−3, 5)	(7 16)	(−21, –14)	(−5, –0.9)
Rajasthan	−30***	−17**	-3	−6**	−4***	−17***	10***	−9***	−15***	−2***
	(−42, –18)	(−30, –4)	(−11, 5)	(−12, –0.5)	(−5, –2)	(−19, –14)	(7, 13)	(−13, –6)	(−18, –12)	(−3, –0.9)
Sikkim			−14***	-2	1			−6**	−9***	6***
			(−23, –5)	(−5, 2)	(−4, 7)			(−11, –0.5)	(−13, –6)	(2, 10)
Tamil Nadu	−6***	−5**	0	−0.8	0.6	−18***	4**	−4*	−18***	−0.6
	(−10, –2)	(−10, –0.6)	(−4, 4)	(−3, 2)	(−1, 2)	(−21, –15)	(0.1, 8)	(−8, 0.7)	(−21, –15)	(−2, 0.9)
Telangana	−10**	-5	0.6	−3*	-2	−4***	−4*	−0.1	−0.3	−0.2
	(−17, –2)	(−14, 4)	(−5, 6)	(−8, 0.6)	(−5, 0.6)	(−7, –1)	(−8, 0.5)	(−4, 4)	(−3, 2)	(−2, 2)
Tripura	−8*	-3	2	−10***	2	−25***	5	−14***	−21***	5***
	(−16, 0.3)	(−15, 10)	(−9, 13)	(−15, –4)	(−1, 6)	(−31, –19)	(−4, 14)	(−23, –6)	(−27, –15)	(1, 9)
Uttar Pradesh	-5	11*	−12**	−4**	−0.5	−9***	3**	-2	−5***	−5***
	(−14, 3)	(−0.4, 23)	(−21, –2)	(−9, –0.3)	(−1.5, 0.4)	(−10, –7)	(0.5, 6)	(−4, 0.6)	(−7, –3)	(−6, –4)
Uttarakhand	−22***	1	−16*	0.1	−7***	-2	9**	-3	−6***	−2*
	(−35, –9)	(−20, 22)	(−34, 2)	(−6, 6)	(−10, –4)	(−5, 2)	(2, 17)	(−10, 5)	(−10, –2)	(−5, 0.4)
West Bengal	−6**	0.8	-3	−6***	2**	−13***	-3	2	−12***	−0.2
	(−10, –1)	(−5, 7)	(−8, 3)	(−10, –3)	(0.4, 4)	(−16, –10)	(−7, 1)	(−2, 6)	(−15, –9)	(−2, 2)
Union territories:										
Andaman & Nico-					1					5**
bar Islands					(−5, 7)					(0.4, 10)
Chandigarh					-5					-3
					(−15, 4)					(−10, 4)
Dadra & Nagar Haveli					−0.8					5*
& Daman & Diu					(−7, 5)					(−0.4, 9)
Jammu & Kashmir	−22***	-4	-9	−5*	−3***	−17***	7***	2	−21***	−5***
	(−32, –12)	(−18, 9)	(−20, 2)	(−12, 1.0)	(−5, –2)	(−19, –14)	(3, 11)	(−4, 7)	(−24, –17)	(−6, –3)
Ladakh					2					-2
					(−3, 8)					(−5, 2)
Lakshadweep					−9**					−14***
					(−16, –1)					(−20, –8)
Nct of Delhi	−5**	-1	0.3	0.1	-4	−7***	−4*	−7***	−0.6	4**
	(−10, –1.0)	(−7, 5)	(−6, 7)	(−7, 7)	(−10, 1)	(−10, –4)	(−7, 0.0)	(−11, –3)	(−4, 3)	(0.9, 7)
Puducherry					-2					2
					(−7, 3)					(−2, 6)

Notes: *p<0.1; **p<0.05; ***p<0.01. 95% confidence intervals are shown below estimates. The colours represent quintiles. Estimates are rounded to whole numbers (except numbers below 1 and when confidence intervals would overlap).

Low birth weight quintiles:
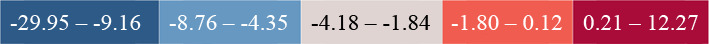

Small at birth quintiles:
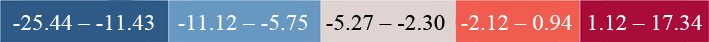

States starting with a high prevalence in 1993 experienced a greater pp change between the 1993 and 2021 surveys, or a Pearson’s correlation coefficient of −0.85 (p<0.01) for low birth weight and −0.89 (p<0.01) for small birth size as assessed by the mother (figure 2).

### Sensitivity and supplementary analyses

Imputing missing values resulted in a much higher prevalence of low birth weight being observed in India overall in the earlier surveys (online supplemental table 4). For example, in the 1993 survey, the overall prevalence was 26% in our main analysis while it increased to 33% after imputing missing birth weight information. The difference was also large for the 1999 and 2006 surveys. The prevalence in some states was more than triple compared with that observed in the main analysis. After imputing missing values, the pp change in prevalence of low birth weight was almost double what was observed in the main analysis in India overall (online supplemental table 5). The correlation between low birth weight prevalence in 1993 and the pp change between 1993 and 2021 was considerably lower than in the main analyses, or a correlation coefficient of −0.53 compared with −0.85 (online supplemental figure 2).

The selection models also showed a higher prevalence in the earlier surveys than our main analysis (online supplemental table 6). For example, in India overall in 1993, the prevalence was 32% (compared with 26% in our main analysis). Correspondingly, the overall pp change across surveys was larger (online supplemental table 7). However, the state-level correlation between prevalence in 1993 and pp change between 1993 and 2021 were similar after estimating prevalence using selection models, as in our main analysis (online supplemental figure 4).

Reweighting the samples to match the socioeconomic distribution in the latest survey indicated a considerably greater prevalence in the second and third surveys, compared with the original analysis (online supplemental table 8) and also increased the observed decline over time (online supplemental table 9). After reweighting the data, the correlation between baseline prevalence and change was similar to that observed in the main analysis (online supplemental figure 6).

Overall, in India according to the 2021 survey, there were 4.2 million low-birth-weight births in a single year (online supplemental table 10). The greatest number of low birth weight births was in Utter Pradesh (858 000), Bihar (430 000), Maharashtra (399 000), and West Bengal (318 000), with these four states accounting for 47% of all low-birth-weight births in India. There were 2.5 million children who were born smaller than average as assessed by the mother, in India overall. The largest number of children with small birth size was also in Uttar Pradesh (462 000), Bihar (318 000), Maharashtra (261 000), and West Bengal (208 000), with these four states accounting for 50% of all children born smaller than average. Finally, states with a high prevalence of low birth weight also tended to have a greater headcount of low-birth-weight births (online supplemental figure 7)**.**

There was a socioeconomic patterning of low birth weight and small birth size in the 2021 survey, where prevalence was considerably greater for children born to women without education or only primary education and children in the poorest households (online supplemental table 11). Females also had greater prevalence than males.

## Discussion

Drawing from the data collated across five waves of the NFHS (1993 to 2021), our results present prevalence of low birth weight in the states and union territories of India. The insights from data spanning nearly three decades shed light on both promising progress and enduring challenges. Our results point to a general decline in the prevalence of low birth weight and convergence between states over time. However, the levels and specific numbers should be interpreted with caution due to data quality issues, particularly low levels of recorded birth weight in the older surveys. Our findings also suggested that mothers have perceived that the health of their newborns has vastly improved, indicated by a sharp reduction in mothers subjectively assessing that their children were born small.

Recent studies have suggested that India had the highest prevalence and accounted for almost a third of all low birth weight births across 158 countries in 2020.^22^ However, since India is more populous than all but one of the world’s continents and its largest state is more populous than all except seven countries,^21^ reporting only national level prevalence of low birth weight obscures information necessary to tailor and implement policy for improving child health.^23^ For example, states such as Manipur, Mizoram, and Nagaland had a prevalence of low birth weight in 2021 similar to that of Canada, the United Kingdom, and other high-income countries, or 4–7%,^22^ exemplifying successes in ensuring maternal and neonatal health. At the other end of the scale, Punjab and Delhi had the highest prevalence of low birth weight, over 22%, signalling ongoing challenges that may include poor maternal nutrition, limited access to quality healthcare, socioeconomic adversities, or a combination of these and other factors.^24 25^ Further, four of 36 states and union territories — Uttar Pradesh, Bihar, Maharashtra, and West Bengal — accounted for almost half of the low birth weight births in India.

Prevalence measures for a point in time give limited insight into successes in reducing low birth weight prevalence or adverse trends across time. Most health policy implementation and formulation in India are at the state-level^23 26 27^: therefore, identifying states demonstrating recent successes in reducing low birth weight can help to identify policy choices and interventions that have improved maternal and child health. For example, the *Empowered Action Group*, established in 2001, gives special attention to eight socioeconomically disadvantaged states through monitoring and facilitating the achievement of health goals, many concerning maternal and child health.^28^ Four states from the *Empowered Action Group*, Rajasthan, Chhattisgarh, Uttarakhand, and Jharkhand, had the most substantial positive shift in reducing the prevalence of low birth weight, or over 20 percentage points across the period, possibly highlighting the effectiveness of targeted interventions stemming from the *Empowered Action Group* designation. However, while Uttarakhand and Jharkhand had a notable acceleration in the decline between 1999–2006, after the implementation of the *Empowered Action Group* in 2001, Chhattisgarh and Rajasthan witnessed the most substantial declines earlier, in 1999. Further, Bihar, Odisha, and Uttar Pradesh had some of the lowest declines in prevalence, between 1–5 percentage points across the period, despite being designated *Empowered Action Group* states, underscoring the imperative need for a revamped approach to address the persistent adverse exposures causing low birth weight in these states. However, it should be noted that Bihar had a more impressive decline 2006–16, or 13 percentage point decline in prevalence of low birth weight.

Our findings suggest the states that began with the higher prevalence in the earliest survey, such as Jharkhand and Chhattisgarh, tended to experience a larger decline over the period studied. The convergence was considerably larger than for other child anthropometric outcomes from a previous study on Indian states, 1993–2016.^11^ This potential ‘catch-up’ phenomenon might indicate that interventions in these regions, catalysed by the initial dire figures, have been impactful. However, it also emphasises the importance of sustained and contextually relevant interventions, even in states that have shown remarkable progress, to ensure this momentum is maintained.

### Limitations

Several limitations must be acknowledged. State-level variations in health infrastructure and recording practices might influence the reported data as well as unobserved maternal and child health factors. One of the most salient limitations is the low number of newborns being weighed at birth in the earlier surveys, possibly skewing the initial prevalence rates downward. Low birth weight is likely to be more prevalent among non-weighed children since weighing correlates strongly with healthcare infrastructure and being born in a health facility. Also, low socioeconomic status is linked to both lack of access to healthcare and low birth weight. Therefore, the prevalence of low birth weight is likely to be underestimated, particularly in the earlier surveys. Therefore, the true downward trends are likely to be stronger than the trends observed. However, although our sensitivity analyses (where we estimated prevalence and changes over time using multiple imputations, selection models, and reweighting, to adjust for differences in weighing of newborns across surveys) provided vastly different estimates in many cases, they still indicated a general downward trend in prevalence of low birth weight, and convergence across states and union territories. Further, the remarkable decrease in the percentage of children not weighed at birth is a strong indication of improved care of mothers and newborns, and we also found that the mothers’ subjective assessment of the size of their newborns vastly improved.

In addition to the relatively small portion of children weighed at birth, the sample size was also substantially smaller in the earlier surveys: therefore, many of these early state level estimates and changes over time were imprecisely estimated due to small sample sizes.

While the NFHS offers rich data there are inherent limitations. The reliance on maternal recall for a portion of the birth weight data can introduce recall bias, potentially affecting the accuracy of the reported weights.^29^ Mothers who were surveyed long after childbirth might not accurately remember or might be influenced by the child’s subsequent growth patterns, potentially leading to overestimations or underestimations of the prevalence. Inconsistencies in the scales used, variations in the timing of the weighing post-birth (immediately after vs a few hours later), and the potential for human error in recording or reporting can introduce measurement errors into the data. Also, birth weight data has been reported to have notable heaping on certain values (e.g. 2500 g), as well as misclassification between live births and still births.^30^ Furthermore, we were not able to distinguish between preterm and full-term births.^31^ Our second outcome variable, size at birth assessed by the mother, was entirely subjective and dependent on the mother’s frame of reference.

Over the study period, there have been changes in state boundaries and the creation of new states. Such geopolitical alterations can influence the continuity and comparability of data. Stratification was based on states in earlier surveys and not representative below the state level. Therefore, breaking states apart may lead to results not being fully state representative in earlier surveys.^19^

The most recent survey was interrupted by the COVID-19 pandemic, which may also have had an impact on low birth weight prevalence and whether children were weighed at birth. However, according to our data, children who were born after the pandemic disruptions were less than a percentage point more likely to be low birth weight and were almost two percentage points more likely to have been weighed at birth.

Considering these limitations, while our findings present a valuable overview of birth outcomes, they should be interpreted with caution. Efforts to standardise measurement procedures, improve the timing of surveys post-birth, and account for geopolitical changes in future studies could help in refining the accuracy and reliability of the results.

## Conclusion

In conclusion, while strides have been made in improving birth outcomes in India, disparities across states highlight the need for tailored interventions. As India continues improving the health of newborns, recognising and addressing these state-specific challenges and leveraging the lessons from successful regions will be paramount. Future research should further delve into the underlying determinants of these disparities, aiding policymakers in crafting targeted and sustainable solutions.

## Supplementary material

10.1136/bmjgh-2024-016732 online supplemental file 1

## Data Availability

Data are available in a public, open access repository.
