# Supplementary material for: Trends in low birth weight across 36 states and union territories in India, 1993-2021
Source: BMJ Glob Health. 2025 Jun 16;10(6):e016732. doi: 10.1136/bmjgh-2024-016732 (PMC12182034; doi:10.1136/bmjgh-2024-016732)
Supplement: online supplemental file 1 [file bmjgh-10-6-s001.docx]

**Supplement:**

**Trends in low birth weight across 36 states and union territories in India, 1993-2021**

[Table S1: Study sample distribution (%) for size at birth from the five National Family Health Surveys (NFHS), 1993–2021 2](#_Toc196943004)

[Table S2: Percent not weighed by socioeconomic characteristics 3](#_Toc196943005)

[Table S3: Percent female, rural, non-educated, and poorest among weighed and children who were not weighed at birth 4](#_Toc196943006)

[Table S4: Prevalence (%) by survey: Low birth weight, imputed and original 5](#_Toc196943007)

[Table S5: Percentage point (pp) change in prevalence across surveys: Low birth weight, imputed and original 8](#_Toc196943008)

[Figure S1. Summary distribution of state and union territory-level prevalence, 1993-2021: Low birth weight, imputed and original 11](#_Toc196943009)

[Figure S2. Relationship between prevalence (%) in 1993 and percentage points (pp) change for 1993–2021: Low birth weight imputed and original 12](#_Toc196943010)

[Table S6: Prevalence (%) by survey: Selection models for low birth weight and percentage not weighed at birth 14](#_Toc196943011)

[Table S7: Percentage point (pp) change in prevalence across surveys: Selection models for low birth weight and percentage not weighed at birth 17](#_Toc196943012)

[Figure S3. Summary distribution of state and union territory-level prevalence, 1993-2021: Selection models for low birth weight and percentage not weighed at birth 20](#_Toc196943013)

[Figure S4. Relationship between prevalence (%) in 1993 and percentage points (pp) change for 1993–2021: Selection models for low birth weight and percentage not weighed at birth 21](#_Toc196943014)

[Table S8: Prevalence (%) by survey: Low birth weight after reweighting samples to match wave five 23](#_Toc196943015)

[Table S9: Percentage point (pp) change in prevalence across surveys: Low birth weight after reweighting samples to match wave five 26](#_Toc196943016)

[Figure S5. Summary distribution of state and union territory-level prevalence, 1993-2021: Low birth weight after reweighting samples to match wave five 28](#_Toc196943017)

[Figure S6. Relationship between prevalence (%) in 1993 and percentage points (pp) change for 1993–2021: Low birth weight after reweighting samples to match wave five 29](#_Toc196943018)

[Table S10: Estimated headcount and percentage share of low birth weight and small birth size across states and union territories in 2021 31](#_Toc196943019)

[Table S11: Sample distribution of low birth weight and small size at birth and prevalence across demographic and socioeconomic characteristics, 2021, India 33](#_Toc196943020)

[Figure S7. Relationship between headcount and prevalence (%) of low birth weight and small size at birth, 2021 34](#_Toc196943021)

## Table S1: Study sample distribution (%) for size at birth from the five National Family Health Surveys (NFHS), 1993–2021

|  | Very | Larger than |  | Smaller than | Very | Don't |  |
| --- | --- | --- | --- | --- | --- | --- | --- |
|  | large | average | Average | average | small | know | Missing |
|  |  |  |  |  |  |  |  |
| NFHS-1 (1992–93) |  | 6,749 (13.8) | 30,986 (63.6) | 10,550 (21.2) |  | 432 (0.9) | 242 (0.5) |
| NFHS-2 (1998–99) |  | 4,641 (13.9) | 19,895 (61.3) | 6,718 (19.4) | 1,655 (5.0) |  | 117 (0.4) |
| NFHS-3 (2005–06) | 1,916 (4.3) | 9,617 (18.9) | 28,733 (54.5) | 7,277 (14.8) | 3,103 (6.0) | 823 (1.3) | 86 (0.2) |
| NFHS-4 (2015–16) | 12,973 (6.2) | 31,121 (12.9) | 177,739 (67.1) | 23,401 (9.0) | 7,979 (3.1) | 6,256 (1.6) | 158 (0.1) |
| NFHS-5 (2019–21) | 15,388 (7.0) | 26,566 (11.7) | 163,929 (69.5) | 18,090 (8.0) | 5,402 (2.6) | 3,545 (1.2) |  |

Number of observations are shown with weighted percentages in parentheses.

## Table S2: Percent not weighed by socioeconomic characteristics

|  | NFHS1 | NFHS2 | NFHS3 | NFHS4 | NFHS5 |
| --- | --- | --- | --- | --- | --- |
|  |  |  |  |  |  |
| Child sex: |  |  |  |  |  |
| Male | 84.3 [83.1, 85.3] | 69.9 [68.4, 71.4] | 59.8 [58.0, 61.5] | 18.6 [18.1, 19.1] | 7.3 [7.0, 7.6] |
| Female | 84.6 [83.4, 85.7] | 71.0 [69.5, 72.5] | 62.5 [60.8, 64.2] | 19.9 [19.4, 20.4] | 7.7 [7.5, 8.1] |
| Place of residence: |  |  |  |  |  |
| Urban | 56.6 [53.8, 59.5] | 40.5 [37.5, 43.5] | 32.4 [29.6, 35.3] | 9.4 [8.8, 10.1] | 4.4 [4.0, 4.9] |
| Rural | 91.9 [91.2, 92.6] | 79.0 [77.7, 80.3] | 70.8 [69.2, 72.4] | 23.0 [22.5, 23.6] | 8.6 [8.3, 8.9] |
| Mother's level of education: |  |  |  |  |  |
| No Education | 95.7 [95.2, 96.2] | 88.3 [87.4, 89.2] | 82.2 [80.9, 83.4] | 37.6 [36.8, 38.3] | 17.5 [16.8, 18.2] |
| Primary | 79.6 [77.8, 81.4] | 67.5 [65.3, 69.6] | 61.2 [59.0, 63.4] | 22.0 [21.2, 22.8] | 10.0 [9.4, 10.6] |
| Secondary | 53.9 [51.7, 56.2] | 46.1 [44.0, 48.2] | 35.4 [33.6, 37.3] | 9.7 [9.4, 10.1] | 4.5 [4.3, 4.7] |
| Higher | 18.7 [15.1, 23.1] | 21.5 [19.1, 24.0] | 7.7 [6.2, 9.6] | 4.1 [3.7, 4.5] | 1.7 [1.5, 2.0] |
| Household wealth quintile: |  |  |  |  |  |
| Poorest | 98.1 [97.6, 98.4] | 91.2 [90.0, 92.2] | 85.8 [84.4, 87.1] | 38.3 [37.5, 39.2] | 15.8 [15.1, 16.4] |
| Poorer | 95.5 [94.8, 96.2] | 85.3 [83.9, 86.6] | 76.4 [74.5, 78.1] | 22.0 [21.4, 22.7] | 8.1 [7.7, 8.5] |
| Middle | 90.4 [89.2, 91.4] | 73.8 [71.8, 75.6] | 60.8 [58.6, 63.1] | 13.3 [12.7, 13.8] | 4.9 [4.6, 5.3] |
| Richer | 79.3 [77.6, 80.8] | 55.6 [53.2, 57.9] | 42.5 [40.2, 44.9] | 7.9 [7.5, 8.4] | 3.1 [2.8, 3.4] |
| Richest | 42.4 [39.7, 45.2] | 30.6 [28.4, 32.9] | 16.7 [15.0, 18.6] | 4.0 [3.7, 4.4] | 2.1 [1.8, 2.4] |

Notes: 95% confidence intervals are shown in square brackets next to estimates. National Family Health Surveys (NFHS).

## Table S3: Percent female, rural, non-educated, and poorest among weighed and children who were not weighed at birth

|  | NFHS1 | NFHS2 | NFHS3 | NFHS4 | NFHS5 |
| --- | --- | --- | --- | --- | --- |
|  |  |  |  |  |  |
| Female: |  |  |  |  |  |
| -Weighed | 48.3 [47.0, 49.7] | 47.3 [46.1, 48.5] | 46.1 [45.3, 46.9] | 47.4 [47.1, 47.7] | 47.9 [47.6, 48.2] |
| -Not weighed | 49.0 [48.4, 49.6] | 48.5 [47.8, 49.3] | 49.0 [48.3, 49.8] | 49.4 [48.9, 50.0] | 49.7 [48.7, 50.6] |
| Rural: |  |  |  |  |  |
| -Weighed | 40.8 [37.5, 44.2] | 55.4 [52.2, 58.5] | 56.0 [53.3, 58.6] | 68.5 [67.5, 69.5] | 72.5 [71.6, 73.3] |
| -Not weighed | 85.7 [84.2, 87.2] | 87.3 [85.7, 88.7] | 86.6 [84.7, 88.3] | 86.3 [85.3, 87.2] | 84.2 [82.5, 85.8] |
| Mother has no education: |  |  |  |  |  |
| -Weighed | 18.4 [16.8, 20.1] | 21.5 [20.2, 22.9] | 22.9 [21.7, 24.1] | 23.2 [22.8, 23.7] | 19.1 [18.7, 19.5] |
| -Not weighed | 75.4 [74.3, 76.4] | 68.1 [66.9, 69.2] | 67.4 [65.9, 68.8] | 58.7 [57.9, 59.5] | 49.8 [48.4, 51.1] |
| Poorest household wealth quintile: |  |  |  |  |  |
| -Weighed | 2.9 [2.4, 3.5] | 6.9 [6.1, 7.9] | 9.3 [8.5, 10.2] | 19.4 [18.9, 19.8] | 22.4 [21.9, 22.9] |
| -Not weighed | 26.9 [25.7, 28.2] | 30.1 [28.7, 31.5] | 35.8 [34.1, 37.5] | 50.6 [49.6, 51.6] | 51.7 [50.2, 53.2] |

Notes: 95% confidence intervals are shown in square brackets next to estimates. National Family Health Surveys (NFHS).

## Table S4: Prevalence (%) by survey: Low birth weight, imputed and original

|  | Low birth weight (imputed) | Low birth weight (original) |
| --- | --- | --- |
| \|  \| \| --- \| \|  \| \|  \| \|  \| \| India \| \|  \| \|  \| \| States: \| \| Andhra Pradesh \| \|  \| \| Arunachal Pradesh \| \|  \| \| Assam \| \|  \| \| Bihar \| \|  \| \| Chhattisgarh \| \|  \| \| Goa \| \|  \| \| Gujarat \| \|  \| \| Haryana \| \|  \| \| Himachal Pradesh \| \|  \| \| Jharkhand \| \|  \| \| Karnataka \| \|  \| \| Kerala \| \|  \| \| Madhya Pradesh \| \|  \| \| Maharashtra \| \|  \| \| Manipur \| \|  \| \| Meghalaya \| \|  \| \| Mizoram \| \|  \| \| Nagaland \| \|  \| \| Odisha \| \|  \| \| Punjab \| \|  \| \| Rajasthan \| \|  \| \| Sikkim \| \|  \| \| Tamil Nadu \| \|  \| \| Telangana \| \|  \| \| Tripura \| \|  \| \| Uttar Pradesh \| \|  \| \| Uttarakhand \| \|  \| \| West Bengal \| \|  \| \|  \| \| Union territories: \| \| Andaman & Nico- \| \| bar Islands \| \| Chandigarh \| \|  \| \| Dadra & Nagar Haveli \| \| & Daman & Diu \| \| Jammu & Kashmir \| \|  \| \| Ladakh \| \|  \| \| Lakshadweep \| \|  \| \| Nct of Delhi \| \|  \| \| Puducherry \| \|  \| | \| 1992 \| 1998 \| 2005 \| 2015 \| 2019 \| \| --- \| --- \| --- \| --- \| --- \| \| –93 \| –99 \| –06 \| –16 \| –21 \| \|  \|  \|  \|  \|  \| \|  \|  \|  \|  \|  \| \| 33 \| 35 \| 30 \| 21.6 \| 19.8 \| \| [28, 39] \| [31, 38] \| [29, 31] \| [21.3, 21.9] \| [19.5, 20.0] \| \|  \|  \|  \|  \|  \| \|  \|  \|  \|  \|  \| \| 34 \| 28 \| 23 \| 18 \| 16 \| \| [27, 42] \| [23, 34] \| [20, 27] \| [17, 20] \| [15, 18] \| \| 33 \| 32 \| 33 \| 21 \| 15 \| \| [27, 40] \| [26, 38] \| [29, 37] \| [18, 24] \| [13, 17] \| \| 31 \| 35 \| 32 \| 20 \| 18 \| \| [25, 38] \| [30, 40] \| [28, 35] \| [18, 21] \| [17, 19] \| \| 31 \| 35 \| 33 \| 22 \| 21 \| \| [25, 38] \| [31, 40] \| [30, 36] \| [21, 23] \| [20, 22] \| \| 31 \| 33 \| 29 \| 16 \| 17 \| \| [22, 42] \| [25, 42] \| [26, 32] \| [15, 17] \| [16, 18] \| \| 34 \| 31 \| 24 \| 23 \| 14 \| \| [31, 38] \| [25, 38] \| [21, 28] \| [18, 28] \| [11, 19] \| \| 29 \| 31 \| 29 \| 20 \| 19 \| \| [24, 34] \| [26, 35] \| [26, 32] \| [19, 21] \| [18, 20] \| \| 38 \| 33 \| 33 \| 22 \| 21 \| \| [32, 44] \| [28, 40] \| [30, 37] \| [21, 24] \| [20, 22] \| \| 38 \| 38 \| 31 \| 22 \| 16 \| \| [33, 43] \| [34, 43] \| [27, 35] \| [20, 24] \| [14, 19] \| \| 33 \| 38 \| 33 \| 20 \| 18 \| \| [24, 44] \| [31, 45] \| [30, 36] \| [18, 21] \| [17, 19] \| \| 32 \| 31 \| 26 \| 18 \| 16 \| \| [28, 38] \| [27, 35] \| [23, 28] \| [16, 19] \| [15, 18] \| \| 27 \| 21 \| 17 \| 16 \| 16 \| \| [24, 30] \| [18, 25] \| [14, 20] \| [14, 17] \| [15, 18] \| \| 34 \| 43 \| 33 \| 24 \| 21.5 \| \| [29, 40] \| [38, 49] \| [30, 36] \| [23, 25] \| [20.6, 22.4] \| \| 34 \| 33 \| 25 \| 20 \| 21 \| \| [31, 38] \| [30, 37] \| [23, 28] \| [19, 22] \| [19, 22] \| \| 28 \| 24 \| 23 \| 16 \| 12 \| \| [21, 36] \| [19, 29] \| [21, 26] \| [15, 17] \| [10, 13] \| \| 33 \| 33 \| 28 \| 19 \| 16 \| \| [27, 40] \| [28, 39] \| [23, 33] \| [17, 21] \| [14, 17] \| \| 18 \| 16 \| 12 \| 9 \| 6 \| \| [13, 25] \| [12, 22] \| [9, 15] \| [7, 11] \| [5, 8] \| \| 31 \| 27 \| 30 \| 22 \| 17 \| \| [23, 42] \| [21, 34] \| [27, 34] \| [20, 24] \| [15, 20] \| \| 35 \| 34 \| 30 \| 22 \| 20 \| \| [29, 42] \| [31, 39] \| [27, 33] \| [21, 23] \| [18, 21] \| \| 36 \| 32 \| 34 \| 19 \| 23 \| \| [30, 42] \| [28, 37] \| [30, 38] \| [17, 20] \| [21, 24] \| \| 36 \| 43 \| 35 \| 24 \| 19 \| \| [29, 43] \| [37, 49] \| [32, 38] \| [23, 25] \| [18, 20] \| \|  \| 31 \| 20 \| 9 \| 11 \| \|  \| [26, 38] \| [16, 26] \| [7, 11] \| [7, 16] \| \| 35 \| 24 \| 19 \| 17 \| 17 \| \| [31, 39] \| [21, 27] \| [17, 22] \| [16, 18] \| [16, 18] \| \| 30 \| 27 \| 23 \| 16 \| 14 \| \| [24, 36] \| [21, 33] \| [20, 27] \| [14, 19] \| [13, 16] \| \| 41 \| 43 \| 34 \| 20 \| 22 \| \| [35, 47] \| [36, 50] \| [29, 39] \| [18, 23] \| [20, 24] \| \| 33 \| 39 \| 33 \| 27 \| 22.3 \| \| [27, 41] \| [34, 43] \| [31, 36] \| [26, 28] \| [21.7, 22.9] \| \| 33 \| 38 \| 33 \| 28 \| 20 \| \| [25, 42] \| [30, 46] \| [30, 36] \| [27, 30] \| [18, 22] \| \| 33 \| 33 \| 31 \| 19 \| 20 \| \| [28, 38] \| [29, 38] \| [28, 34] \| [18, 20] \| [18, 21] \| \|  \|  \|  \|  \|  \| \|  \|  \|  \|  \|  \| \|  \|  \|  \| 17 \| 18 \| \|  \|  \|  \| [13, 21] \| [14, 22] \| \|  \|  \|  \| 23 \| 17 \| \|  \|  \|  \| [16, 31] \| [11, 25] \| \|  \|  \|  \| 23 \| 22 \| \|  \|  \|  \| [19, 28] \| [18, 26] \| \| 37 \| 41 \| 38 \| 20 \| 13 \| \| [31, 44] \| [36, 47] \| [33, 43] \| [19, 22] \| [11, 14] \| \|  \|  \|  \| 15 \| 13 \| \|  \|  \|  \| [12, 19] \| [10, 18] \| \|  \|  \|  \| 18 \| 10 \| \|  \|  \|  \| [13, 26] \| [6, 14] \| \| 36 \| 32 \| 29 \| 28 \| 23 \| \| [32, 42] \| [27, 37] \| [26, 33] \| [23, 32] \| [21, 25] \| \|  \|  \|  \| 16 \| 14 \| \|  \|  \|  \| [13, 19] \| [10, 19] \| | \| 1992 \| 1998 \| 2005 \| 2015 \| 2019 \| \| --- \| --- \| --- \| --- \| --- \| \| –93 \| –99 \| –06 \| –16 \| –21 \| \|  \|  \|  \|  \|  \| \|  \|  \|  \|  \|  \| \| 26 \| 23 \| 21.5 \| 18.2 \| 18.2 \| \| [24, 27] \| [21, 24] \| [20.7, 22.4] \| [17.9, 18.5] \| [18.0, 18.5] \| \|  \|  \|  \|  \|  \| \|  \|  \|  \|  \|  \| \| 28 \| 18 \| 19 \| 18 \| 16 \| \| [20, 37] \| [13, 24] \| [16, 23] \| [16, 19] \| [15, 18] \| \| 20 \| 25 \| 14 \| 11 \| 11 \| \| [13, 29] \| [18, 34] \| [10, 19] \| [9, 12] \| [9, 12] \| \| 22 \| 33 \| 19 \| 16 \| 16 \| \| [15, 31] \| [26, 42] \| [15, 24] \| [15, 17] \| [15, 17] \| \| 18 \| 15 \| 28 \| 14.4 \| 17 \| \| [12, 25] \| [10, 23] \| [22, 34] \| [13.7, 15.1] \| [16, 18] \| \| 42 \| 28 \| 17 \| 13 \| 16 \| \| [27, 60] \| [16, 45] \| [13, 22] \| [12, 14] \| [15, 17] \| \| 25 \| 25 \| 22 \| 22 \| 14 \| \| [21, 30] \| [19, 32] \| [19, 26] \| [18, 27] \| [10, 19] \| \| 21 \| 20 \| 22 \| 19 \| 19 \| \| [18, 25] \| [16, 24] \| [19, 25] \| [18, 20] \| [17, 20] \| \| 26 \| 24 \| 33 \| 20 \| 21 \| \| [18, 36] \| [19, 31] \| [28, 38] \| [19, 22] \| [19, 22] \| \| 28 \| 35 \| 25 \| 20 \| 16 \| \| [21, 36] \| [29, 42] \| [21, 29] \| [18, 22] \| [14, 18] \| \| 41 \| 37 \| 19 \| 14.5 \| 16 \| \| [32, 49] \| [24, 53] \| [15, 24] \| [13.6, 15.5] \| [15, 17] \| \| 21 \| 17 \| 19 \| 17 \| 16 \| \| [17, 27] \| [14, 22] \| [17, 21] \| [16, 19] \| [15, 17] \| \| 18 \| 18 \| 16 \| 15 \| 16 \| \| [16, 21] \| [15, 21] \| [13, 20] \| [14, 17] \| [15, 18] \| \| 35 \| 33 \| 23 \| 22 \| 20.5 \| \| [29, 41] \| [26, 41] \| [20, 27] \| [21, 23] \| [19.7, 21.4] \| \| 32 \| 26 \| 22 \| 20 \| 20 \| \| [28, 36] \| [23, 30] \| [20, 25] \| [18, 21] \| [19, 21] \| \| 27 \| 10 \| 13 \| 9 \| 7 \| \| [18, 37] \| [7, 15] \| [11, 16] \| [8, 10] \| [6, 9] \| \| 19 \| 15 \| 18 \| 12 \| 12 \| \| [14, 26] \| [10, 22] \| [14, 23] \| [11, 14] \| [10, 13] \| \| 6 \| 9 \| 8 \| 6 \| 4 \| \| [3, 10] \| [6, 13] \| [6, 10] \| [5, 8] \| [3, 5] \| \| 11 \| 8 \| 11 \| 8 \| 5 \| \| [1, 53] \| [2, 30] \| [7, 16] \| [6, 10] \| [4, 6] \| \| 23 \| 25 \| 21 \| 21 \| 19 \| \| [16, 32] \| [19, 32] \| [17, 24] \| [20, 22] \| [18, 20] \| \| 29 \| 24 \| 28 \| 17 \| 22 \| \| [21, 38] \| [18, 31] \| [23, 32] \| [16, 19] \| [21, 24] \| \| 48 \| 30 \| 27 \| 21 \| 18 \| \| [36, 59] \| [25, 36] \| [22, 33] \| [20, 22] \| [17, 19] \| \|  \| 24 \| 10 \| 8 \| 10 \| \|  \| [17, 33] \| [8, 14] \| [7, 11] \| [6, 16] \| \| 23 \| 17 \| 17 \| 16 \| 17 \| \| [19, 27] \| [15, 20] \| [15, 20] \| [15, 17] \| [16, 18] \| \| 24 \| 19 \| 19 \| 16 \| 14 \| \| [17, 32] \| [15, 24] \| [16, 23] \| [14, 18] \| [13, 15] \| \| 28 \| 25 \| 27 \| 17 \| 20 \| \| [20, 36] \| [16, 36] \| [22, 33] \| [15, 20] \| [18, 22] \| \| 25 \| 37 \| 25 \| 20.7 \| 20.2 \| \| [18, 35] \| [29, 45] \| [21, 29] \| [20.0, 21.4] \| [19.5, 20.8] \| \| 40 \| 41 \| 25 \| 25 \| 18 \| \| [28, 53] \| [26, 58] \| [20, 30] \| [22, 27] \| [16, 20] \| \| 25 \| 25 \| 23 \| 17 \| 19 \| \| [21, 29] \| [21, 30] \| [20, 26] \| [15, 18] \| [18, 20] \| \|  \|  \|  \|  \|  \| \|  \|  \|  \|  \|  \| \|  \|  \|  \| 16 \| 17 \| \|  \|  \|  \| [12, 21] \| [13, 22] \| \|  \|  \|  \| 22 \| 17 \| \|  \|  \|  \| [16, 30] \| [11, 24] \| \|  \|  \|  \| 22 \| 21 \| \|  \|  \|  \| [18, 27] \| [17, 25] \| \| 33 \| 28 \| 19 \| 14 \| 11 \| \| [24, 43] \| [20, 38] \| [14, 26] \| [13, 15] \| [10, 12] \| \|  \|  \|  \| 9 \| 12 \| \|  \|  \|  \| [6, 13] \| [8, 16] \| \|  \|  \|  \| 18 \| 10 \| \|  \|  \|  \| [13, 26] \| [6, 14] \| \| 27 \| 26 \| 26 \| 27 \| 22 \| \| [24, 31] \| [22, 31] \| [22, 31] \| [22, 32] \| [20, 24] \| \|  \|  \|  \| 16 \| 14 \| \|  \|  \|  \| [13, 19] \| [10, 19] \| |

| Low birth weight (imputed) quintiles: | Low birth weight (original) quintiles: |
| --- | --- |
| \| 6.35 – 17.03 \| 17.07 – 21.79 \| 21.80 – 28.88 \| 28.93 – 33.16 \| 33.29 – 43.36 \| \| --- \| --- \| --- \| --- \| --- \| | \| 4.02 – 14.14 \| 14.40 – 17.65 \| 17.67 – 20.76 \| 20.84 – 25.31 \| 25.36 – 47.66 \| \| --- \| --- \| --- \| --- \| --- \| |

Notes: 95% confidence intervals are shown in square brackets below estimates. The colors represent quintiles. 'Imputed' refers to results obtained after imputing birth weight for children without a birth weight measure using measures of demographic and socioeconomic characteristics. Estimates are rounded to whole numbers (except numbers below 1 and when confidence intervals would overlap).

## Table S5: Percentage point (pp) change in prevalence across surveys: Low birth weight, imputed and original

|  | Low birth weight (imputed) | Low birth weight (original) |
| --- | --- | --- |
| \|  \| \| --- \| \|  \| \|  \| \|  \| \| India \| \|  \| \|  \| \| States: \| \| Andhra Pradesh \| \|  \| \| Arunachal Pradesh \| \|  \| \| Assam \| \|  \| \| Bihar \| \|  \| \| Chhattisgarh \| \|  \| \| Goa \| \|  \| \| Gujarat \| \|  \| \| Haryana \| \|  \| \| Himachal Pradesh \| \|  \| \| Jharkhand \| \|  \| \| Karnataka \| \|  \| \| Kerala \| \|  \| \| Madhya Pradesh \| \|  \| \| Maharashtra \| \|  \| \| Manipur \| \|  \| \| Meghalaya \| \|  \| \| Mizoram \| \|  \| \| Nagaland \| \|  \| \| Odisha \| \|  \| \| Punjab \| \|  \| \| Rajasthan \| \|  \| \| Sikkim \| \|  \| \| Tamil Nadu \| \|  \| \| Telangana \| \|  \| \| Tripura \| \|  \| \| Uttar Pradesh \| \|  \| \| Uttarakhand \| \|  \| \| West Bengal \| \|  \| \|  \| \| Union territories: \| \| Andaman & Nico- \| \| bar Islands \| \| Chandigarh \| \|  \| \| Dadra & Nagar Haveli \| \| & Daman & Diu \| \| Jammu & Kashmir \| \|  \| \| Ladakh \| \|  \| \| Lakshadweep \| \|  \| \| Nct of Delhi \| \|  \| \| Puducherry \| \|  \| | \| 1992/3– \| 1992/3– \| 1998/9– \| 2005/6– \| 2015/6– \| \| --- \| --- \| --- \| --- \| --- \| \| 2019/21 \| 1998/9 \| 2005/6 \| 2015/6 \| 2019/21 \| \|  \|  \|  \|  \|  \| \|  \|  \|  \|  \|  \| \| -13*** \| 1 \| -4** \| -9*** \| -1.8*** \| \| [-19, -8] \| [-5, 8] \| [-8, -0.6] \| [-10, -7] \| [-2.2, -1.4] \| \|  \|  \|  \|  \|  \| \|  \|  \|  \|  \|  \| \| -18*** \| -6 \| -5 \| -5*** \| -2 \| \| [-25, -10] \| [-16, 3] \| [-11, 2] \| [-9, -2] \| [-4, 0.7] \| \| -18*** \| -1 \| 0.8 \| -12*** \| -6*** \| \| [-25, -11] \| [-10, 8] \| [-6, 8] \| [-17, -7] \| [-9, -3] \| \| -14*** \| 3 \| -3 \| -12*** \| -2** \| \| [-20, -7] \| [-4, 11] \| [-10, 4] \| [-16, -8] \| [-4, -0.4] \| \| -10*** \| 4 \| -2 \| -11*** \| -1* \| \| [-17, -4] \| [-4, 12] \| [-8, 4] \| [-13, -8] \| [-2, 0.0] \| \| -14*** \| 2 \| -5 \| -13*** \| 1.0 \| \| [-24, -4] \| [-13, 17] \| [-14, 5] \| [-16, -9] \| [-0.8, 3] \| \| -20*** \| -3 \| -7* \| -1 \| -9*** \| \| [-25, -15] \| [-11, 5] \| [-15, 0.8] \| [-7, 4] \| [-15, -2] \| \| -10*** \| 2 \| -2 \| -9*** \| -1 \| \| [-15, -5] \| [-5, 8] \| [-7, 4] \| [-12, -6] \| [-3, 0.5] \| \| -17*** \| -4 \| 0.0 \| -11*** \| -1 \| \| [-22, -11] \| [-13, 4] \| [-7, 7] \| [-15, -7] \| [-3, 0.9] \| \| -21*** \| 0.5 \| -7** \| -9*** \| -5*** \| \| [-26, -16] \| [-6, 7] \| [-13, -2] \| [-13, -4] \| [-9, -2] \| \| -15*** \| 5 \| -5 \| -14*** \| -1* \| \| [-25, -4] \| [-8, 18] \| [-12, 3] \| [-17, -10] \| [-3, 0.1] \| \| -16*** \| -2 \| -5** \| -8*** \| -2* \| \| [-21, -11] \| [-9, 6] \| [-9, -0.9] \| [-10, -5] \| [-3, 0.3] \| \| -10*** \| -6** \| -4* \| -1 \| 0.8 \| \| [-14, -7] \| [-10, -1] \| [-9, 0.5] \| [-5, 2] \| [-2, 3] \| \| -13*** \| 9** \| -11*** \| -9*** \| -3*** \| \| [-19, -7] \| [0.3, 18] \| [-17, -4] \| [-12, -5] \| [-4, -1] \| \| -14*** \| -1 \| -8*** \| -5*** \| 0.5 \| \| [-18, -10] \| [-6, 4] \| [-12, -4] \| [-8, -2] \| [-1, 2] \| \| -17*** \| -4 \| -0.5 \| -8*** \| -4*** \| \| [-25, -9] \| [-14, 6] \| [-6, 5] \| [-11, -5] \| [-6, -2] \| \| -18*** \| -0.2 \| -5 \| -9*** \| -4** \| \| [-24, -11] \| [-9, 9] \| [-13, 2] \| [-14, -3] \| [-6, -0.7] \| \| -12*** \| -2 \| -5 \| -3 \| -3** \| \| [-18, -6] \| [-10, 6] \| [-10, 1] \| [-6, 0.7] \| [-5, -0.3] \| \| -14*** \| -5 \| 3 \| -8*** \| -5*** \| \| [-24, -5] \| [-16, 7] \| [-4, 11] \| [-12, -5] \| [-8, -2] \| \| -16*** \| -0.7 \| -5* \| -8*** \| -2*** \| \| [-22, -9] \| [-9, 8] \| [-10, 0.1] \| [-11, -5] \| [-4, -0.7] \| \| -13*** \| -4 \| 1 \| -15*** \| 4*** \| \| [-19, -6] \| [-13, 6] \| [-4, 7] \| [-19, -11] \| [2, 7] \| \| -17*** \| 7 \| -8** \| -11*** \| -5*** \| \| [-24, -10] \| [-5, 18] \| [-15, -1] \| [-14, -8] \| [-7, -4] \| \|  \|  \| -11** \| -12*** \| 2 \| \|  \|  \| [-19, -2] \| [-17, -6] \| [-3, 7] \| \| -18*** \| -11*** \| -4** \| -3** \| 0.4 \| \| [-22, -13] \| [-16, -6] \| [-8, -0.2] \| [-5, -0.2] \| [-1, 2] \| \| -16*** \| -3 \| -3 \| -7*** \| -2* \| \| [-22, -10] \| [-13, 6] \| [-10, 3] \| [-11, -3] \| [-5, 0.4] \| \| -19*** \| 1 \| -9** \| -14*** \| 1 \| \| [-26, -13] \| [-9, 11] \| [-17, -0.3] \| [-19, -8] \| [-2, 5] \| \| -11*** \| 5 \| -6* \| -6*** \| -5*** \| \| [-18, -4] \| [-4, 15] \| [-12, 1.0] \| [-9, -4] \| [-6, -4] \| \| -13*** \| 5 \| -5 \| -4** \| -9*** \| \| [-22, -4] \| [-8, 18] \| [-13, 4] \| [-8, -1] \| [-12, -6] \| \| -13*** \| 0.5 \| -2 \| -12*** \| 0.6 \| \| [-19, -8] \| [-7, 8] \| [-8, 3] \| [-16, -9] \| [-1, 2] \| \|  \|  \|  \|  \|  \| \|  \|  \|  \|  \|  \| \|  \|  \|  \|  \| 1.0 \| \|  \|  \|  \|  \| [-5, 7] \| \|  \|  \|  \|  \| -6 \| \|  \|  \|  \|  \| [-16, 4] \| \|  \|  \|  \|  \| -1 \| \|  \|  \|  \|  \| [-7, 5] \| \| -24*** \| 4 \| -4 \| -18*** \| -7*** \| \| [-31, -18] \| [-5, 14] \| [-10, 3] \| [-23, -13] \| [-9, -5] \| \|  \|  \|  \|  \| -2 \| \|  \|  \|  \|  \| [-7, 4] \| \|  \|  \|  \|  \| -9** \| \|  \|  \|  \|  \| [-16, -1] \| \| -14*** \| -4 \| -3 \| -1 \| -5* \| \| [-19, -8] \| [-10, 1] \| [-9, 3] \| [-7, 4] \| [-10, 0.2] \| \|  \|  \|  \|  \| -2 \| \|  \|  \|  \|  \| [-7, 3] \| | \| 1992/3– \| 1992/3– \| 1998/9– \| 2005/6– \| 2015/6– \| \| --- \| --- \| --- \| --- \| --- \| \| 2019/21 \| 1998/9 \| 2005/6 \| 2015/6 \| 2019/21 \| \|  \|  \|  \|  \|  \| \|  \|  \|  \|  \|  \| \| -8*** \| -3*** \| -1 \| -3*** \| 0.0 \| \| [-9, -6] \| [-5, -1] \| [-3, 0.4] \| [-4, -2] \| [-0.4, 0.4] \| \|  \|  \|  \|  \|  \| \|  \|  \|  \|  \|  \| \| -12*** \| -10* \| 2 \| -2 \| -1 \| \| [-20, -3] \| [-20, 0.2] \| [-5, 8] \| [-6, 2] \| [-4, 0.9] \| \| -9** \| 5 \| -11** \| -3 \| -0.1 \| \| [-18, -0.8] \| [-6, 17] \| [-20, -2] \| [-8, 1] \| [-2, 2] \| \| -6 \| 11* \| -14*** \| -4 \| 0.4 \| \| [-14, 2] \| [-0.0, 23] \| [-24, -4] \| [-8, 1] \| [-1, 2] \| \| -0.9 \| -2 \| 12*** \| -13*** \| 2*** \| \| [-8, 6] \| [-12, 7] \| [3, 22] \| [-19, -7] \| [1, 3] \| \| -26*** \| -15 \| -10 \| -5** \| 3*** \| \| [-44, -9] \| [-37, 8] \| [-26, 5] \| [-9, -0.3] \| [2, 5] \| \| -11*** \| -0.1 \| -3 \| 0.0 \| -8*** \| \| [-17, -6] \| [-8, 8] \| [-10, 4] \| [-6, 6] \| [-14, -2] \| \| -3 \| -1 \| 2 \| -3* \| -0.5 \| \| [-6, 0.8] \| [-7, 4] \| [-3, 7] \| [-6, 0.5] \| [-2, 1] \| \| -5 \| -2 \| 8** \| -12*** \| 0.1 \| \| [-14, 3] \| [-12, 9] \| [0.4, 16] \| [-17, -7] \| [-2, 2] \| \| -12*** \| 8 \| -10** \| -5** \| -4** \| \| [-20, -4] \| [-3, 18] \| [-18, -2] \| [-10, -0.6] \| [-7, -0.9] \| \| -25*** \| -3 \| -18** \| -5* \| 1* \| \| [-34, -16] \| [-20, 14] \| [-34, -3] \| [-9, 0.1] \| [-0.2, 2] \| \| -5** \| -4 \| 1 \| -2 \| -1 \| \| [-11, -0.4] \| [-10, 2] \| [-3, 6] \| [-4, 1] \| [-3, 0.6] \| \| -2 \| -0.6 \| -1 \| -0.6 \| 0.8 \| \| [-5, 1] \| [-4, 3] \| [-6, 3] \| [-4, 3] \| [-2, 3] \| \| -14*** \| -1 \| -10** \| -2 \| -1** \| \| [-21, -8] \| [-11, 8] \| [-18, -2] \| [-5, 2] \| [-2, -0.2] \| \| -12*** \| -6** \| -4* \| -3* \| 0.5 \| \| [-16, -8] \| [-12, -1] \| [-8, 0.5] \| [-5, 0.2] \| [-1, 2] \| \| -19*** \| -16*** \| 3 \| -4*** \| -2** \| \| [-29, -10] \| [-27, -6] \| [-2, 8] \| [-7, -1] \| [-4, -0.2] \| \| -7** \| -4 \| 3 \| -6** \| -0.5 \| \| [-14, -1] \| [-13, 4] \| [-4, 11] \| [-10, -1] \| [-3, 2] \| \| -2 \| 3 \| -1 \| -2 \| -2** \| \| [-5, 2] \| [-2, 7] \| [-5, 3] \| [-4, 1.0] \| [-4, -0.1] \| \| -6 \| -3 \| 2 \| -3 \| -3*** \| \| [-28, 15] \| [-27, 22] \| [-10, 15] \| [-8, 1] \| [-5, -0.9] \| \| -4 \| 2 \| -4 \| 0.2 \| -2** \| \| [-12, 4] \| [-8, 12] \| [-12, 3] \| [-3, 4] \| [-3, -0.1] \| \| -6 \| -5 \| 4 \| -11*** \| 5*** \| \| [-15, 3] \| [-16, 6] \| [-4, 12] \| [-15, -6] \| [3, 7] \| \| -30*** \| -17** \| -3 \| -6** \| -4*** \| \| [-42, -18] \| [-30, -4] \| [-11, 5] \| [-12, -0.5] \| [-5, -2] \| \|  \|  \| -14*** \| -2 \| 1 \| \|  \|  \| [-23, -5] \| [-5, 2] \| [-4, 7] \| \| -6*** \| -5** \| -0.0 \| -0.8 \| 0.6 \| \| [-10, -2] \| [-10, -0.6] \| [-4, 4] \| [-3, 2] \| [-1, 2] \| \| -10** \| -5 \| 0.6 \| -3* \| -2 \| \| [-17, -2] \| [-14, 4] \| [-5, 6] \| [-8, 0.6] \| [-5, 0.6] \| \| -8* \| -3 \| 2 \| -10*** \| 2 \| \| [-16, 0.3] \| [-15, 10] \| [-9, 13] \| [-15, -4] \| [-1, 6] \| \| -5 \| 11* \| -12** \| -4** \| -0.5 \| \| [-14, 3] \| [-0.4, 23] \| [-21, -2] \| [-9, -0.3] \| [-1.5, 0.4] \| \| -22*** \| 1 \| -16* \| 0.1 \| -7*** \| \| [-35, -9] \| [-20, 22] \| [-34, 2] \| [-6, 6] \| [-10, -4] \| \| -6** \| 0.8 \| -3 \| -6*** \| 2** \| \| [-10, -1] \| [-5, 7] \| [-8, 3] \| [-10, -3] \| [0.4, 4] \| \|  \|  \|  \|  \|  \| \|  \|  \|  \|  \|  \| \|  \|  \|  \|  \| 1 \| \|  \|  \|  \|  \| [-5, 7] \| \|  \|  \|  \|  \| -5 \| \|  \|  \|  \|  \| [-15, 4] \| \|  \|  \|  \|  \| -0.8 \| \|  \|  \|  \|  \| [-7, 5] \| \| -22*** \| -4 \| -9 \| -5* \| -3*** \| \| [-32, -12] \| [-18, 9] \| [-20, 2] \| [-12, 1.0] \| [-5, -2] \| \|  \|  \|  \|  \| 2 \| \|  \|  \|  \|  \| [-3, 8] \| \|  \|  \|  \|  \| -9** \| \|  \|  \|  \|  \| [-16, -1] \| \| -5** \| -1 \| 0.3 \| 0.1 \| -4 \| \| [-10, -1.0] \| [-7, 5] \| [-6, 7] \| [-7, 7] \| [-10, 1] \| \|  \|  \|  \|  \| -2 \| \|  \|  \|  \|  \| [-7, 3] \| |

| Low birth weight (imputed) quintiles: | | | | Low birth weight (original) quintiles: |
| --- | --- | --- | --- | --- |
| \| -24.42 – -12.65 \| -12.30 – -7.30 \| -6.95 – -4.17 \| -4.10 – -1.26 \| -1.19 – 9.22 \| \| --- \| --- \| --- \| --- \| --- \| | | | | \| -29.95 – -9.16 \| -8.76 – -4.35 \| -4.18 – -1.84 \| -1.80 – 0.12 \| 0.21 – 12.27 \| \| --- \| --- \| --- \| --- \| --- \| |
|  |  |  |  |  |
|  |  |  |  |  |

Notes: *p<0.1; **p<0.05; p<0.01. 95% confidence intervals are shown in square brackets below estimates. The colors represent quintiles. 'Imputed' refers to results obtained after imputing birth weight for children without a birth weight measure using measures of demographic and socioeconomic characteristics. Estimates are rounded to whole numbers (except numbers below 1 and when confidence intervals would overlap).

## Figure S1. Summary distribution of state and union territory-level prevalence, 1993-2021: Low birth weight, imputed and original

Notes: Percentiles 5 and 95 (line) and 25, 50, and 75 (box) are shown. Dots indicate state estimates. States were equally weighted for the median and percentiles. 'Imputed' refers to results obtained after imputing birth weight for children without a birth weight measure using measures of demographic and socioeconomic characteristics.

## Figure S2. Relationship between prevalence (%) in 1993 and percentage points (pp) change for 1993–2021: Low birth weight imputed and original

Notes: State labels were randomly displaced by at most 10% on either axis for readability. 'Imputed' refers to results obtained after imputing birth weight for children without a birth weight measure using measures of demographic and socioeconomic characteristics. Andhra Pradesh (AP), Arunachal Pradesh (AR), Assam (AS), Bihar (BR), Chhattisgarh (CG), Goa (GA), Gujarat (GJ), Haryana (HR), Himachal Pradesh (HP), Jammu & Kashmir (JK), Jharkhand (JH), Karnataka (KA), Kerala (KL), Madhya Pradesh (MP), Maharashtra (MH), Manipur (MN), Meghalaya (ML), Mizoram (MZ), Nagaland (NL), Nct of Delhi (DL), Odisha (OR), Punjab (PB), Rajasthan (RJ), Tamil Nadu (TN), Telangana (TL), Tripura (TR), Uttar Pradesh (UP), Uttarakhand (UK), West Bengal (WB).

## Table S6: Prevalence (%) by survey: Selection models for low birth weight and percentage not weighed at birth

|  | Low birth weight (selection corrected) | Not weighed at birth |
| --- | --- | --- |
| \|  \| \| --- \| \|  \| \|  \| \|  \| \| India \| \|  \| \|  \| \| States: \| \| Andhra Pradesh \| \|  \| \| Arunachal Pradesh \| \|  \| \| Assam \| \|  \| \| Bihar \| \|  \| \| Chhattisgarh \| \|  \| \| Goa \| \|  \| \| Gujarat \| \|  \| \| Haryana \| \|  \| \| Himachal Pradesh \| \|  \| \| Jharkhand \| \|  \| \| Karnataka \| \|  \| \| Kerala \| \|  \| \| Madhya Pradesh \| \|  \| \| Maharashtra \| \|  \| \| Manipur \| \|  \| \| Meghalaya \| \|  \| \| Mizoram \| \|  \| \| Nagaland \| \|  \| \| Odisha \| \|  \| \| Punjab \| \|  \| \| Rajasthan \| \|  \| \| Sikkim \| \|  \| \| Tamil Nadu \| \|  \| \| Telangana \| \|  \| \| Tripura \| \|  \| \| Uttar Pradesh \| \|  \| \| Uttarakhand \| \|  \| \| West Bengal \| \|  \| \|  \| \| Union territories: \| \| Andaman & Nico- \| \| bar Islands \| \| Chandigarh \| \|  \| \| Dadra & Nagar Haveli \| \| & Daman & Diu \| \| Jammu & Kashmir \| \|  \| \| Ladakh \| \|  \| \| Lakshadweep \| \|  \| \| Nct of Delhi \| \|  \| \| Puducherry \| \|  \| | \| 1992 \| 1998 \| 2005 \| 2015 \| 2019 \| \| --- \| --- \| --- \| --- \| --- \| \| –93 \| –99 \| –06 \| –16 \| –21 \| \|  \|  \|  \|  \|  \| \|  \|  \|  \|  \|  \| \| 32 \| 31 \| 27 \| 18.1 \| 18 \| \| [28, 37] \| [28, 34] \| [25, 29] \| [17.7, 18.4] \| [15, 21] \| \|  \|  \|  \|  \|  \| \|  \|  \|  \|  \|  \| \| 33 \| 20 \| 21 \| 18 \| 16 \| \| [24, 44] \| [15, 26] \| [18, 25] \| [16, 19] \| [14, 18] \| \| 22 \| 28 \| 18 \| 11 \| 10 \| \| [15, 32] \| [21, 36] \| [13, 23] \| [9, 13] \| [6, 16] \| \| 26 \| 38 \| 24 \| 16 \| 16 \| \| [18, 36] \| [30, 47] \| [19, 29] \| [15, 17] \| [13, 19] \| \| 24 \| 21 \| 33 \| 14 \| 16 \| \| [16, 33] \| [15, 30] \| [26, 39] \| [13, 15] \| [11, 23] \| \| 49 \| 32 \| 22 \| 12.5 \| 16 \| \| [32, 66] \| [20, 46] \| [17, 27] \| [11.5, 13.5] \| [14, 18] \| \| 27 \| 27 \| 23 \| 22 \| 14 \| \| [22, 31] \| [21, 33] \| [20, 26] \| [18, 27] \| [11, 18] \| \| 25 \| 23 \| 25 \| 19 \| 18 \| \| [20, 29] \| [19, 28] \| [21, 28] \| [18, 20] \| [17, 20] \| \| 32 \| 29 \| 37 \| 20 \| 20 \| \| [23, 42] \| [22, 36] \| [32, 42] \| [19, 22] \| [19, 22] \| \| 32 \| 38 \| 28 \| 20 \| 16 \| \| [24, 41] \| [31, 45] \| [23, 32] \| [18, 22] \| [13, 18] \| \| 47 \| 43 \| 24 \| 14 \| 15 \| \| [37, 57] \| [29, 59] \| [20, 30] \| [13, 15] \| [11, 20] \| \| 25 \| 21 \| 21 \| 17 \| 16 \| \| [20, 31] \| [17, 25] \| [18, 23] \| [16, 18] \| [14, 17] \| \| 19 \| 18 \| 16 \| 15 \| 16 \| \| [17, 22] \| [15, 21] \| [13, 20] \| [14, 17] \| [15, 18] \| \| 41 \| 39 \| 28 \| 22 \| 20 \| \| [33, 49] \| [31, 47] \| [24, 32] \| [21, 23] \| [18, 23] \| \| 35 \| 29 \| 24 \| 19 \| 20 \| \| [31, 39] \| [25, 33] \| [21, 26] \| [18, 21] \| [18, 22] \| \| 30 \| 13 \| 16 \| 9 \| 7 \| \| [21, 41] \| [9, 18] \| [13, 19] \| [8, 10] \| [4, 12] \| \| 22 \| 18 \| 21 \| 12 \| 11 \| \| [16, 29] \| [12, 26] \| [17, 26] \| [10, 14] \| [7, 17] \| \| 7 \| 10 \| 9 \| 6 \| 4 \| \| [5, 11] \| [7, 13] \| [7, 11] \| [5, 8] \| [2, 7] \| \| 17 \| 12 \| 16 \| 8 \| 3 \| \| [4, 50] \| [5, 27] \| [12, 21] \| [6, 10] \| [0.1, 57] \| \| 27 \| 28 \| 23 \| 21 \| 19 \| \| [20, 37] \| [22, 34] \| [20, 27] \| [20, 22] \| [18, 20] \| \| 34 \| 28 \| 31 \| 17 \| 22 \| \| [25, 44] \| [22, 34] \| [27, 36] \| [16, 19] \| [20, 25] \| \| 55 \| 36 \| 32 \| 21 \| 17 \| \| [41, 68] \| [29, 43] \| [27, 38] \| [20, 22] \| [16, 19] \| \|  \| 27 \| 12 \| 8 \| 10 \| \|  \| [20, 37] \| [10, 16] \| [7, 11] \| [6, 16] \| \| 25 \| 18 \| 18 \| 16 \| 17 \| \| [21, 30] \| [16, 22] \| [16, 20] \| [15, 17] \| [16, 18] \| \| 27 \| 21 \|  \| 16 \| 14 \| \| [20, 36] \| [17, 26] \|  \| [14, 18] \| [12, 15] \| \| 31 \| 28 \| 30 \| 17 \| 19 \| \| [23, 40] \| [19, 38] \| [25, 35] \| [15, 20] \| [16, 23] \| \| 32 \| 44 \| 32 \| 20.4 \| 20 \| \| [23, 42] \| [35, 53] \| [28, 37] \| [19.5, 21.3] \| [16, 24] \| \| 47 \| 49 \| 30 \| 25 \| 17 \| \| [34, 60] \| [34, 65] \| [25, 36] \| [23, 27] \| [14, 22] \| \| 28 \| 28 \| 25 \| 17 \| 19 \| \| [23, 33] \| [24, 34] \| [22, 29] \| [15, 18] \| [17, 21] \| \|  \|  \|  \|  \|  \| \|  \|  \|  \|  \|  \| \|  \|  \|  \| 16 \| 17 \| \|  \|  \|  \| [12, 21] \| [13, 22] \| \|  \|  \|  \| 22 \| 17 \| \|  \|  \|  \| [16, 31] \| [11, 24] \| \|  \|  \|  \| 22 \| 21 \| \|  \|  \|  \| [18, 26] \| [17, 25] \| \| 38 \| 35 \| 26 \| 14 \| 10 \| \| [28, 50] \| [26, 45] \| [21, 32] \| [13, 15] \| [8, 14] \| \|  \|  \|  \| 9 \| 11 \| \|  \|  \|  \| [6, 13] \| [8, 16] \| \|  \|  \|  \| 18 \| 10 \| \|  \|  \|  \| [13, 26] \| [6, 14] \| \| 31 \| 30 \| 30 \| 27 \| 22 \| \| [27, 36] \| [25, 35] \| [26, 35] \| [22, 32] \| [20, 24] \| \|  \|  \|  \| 16 \| 14 \| \|  \|  \|  \| [14, 19] \| [10, 19] \| | \| 1992 \| 1998 \| 2005 \| 2015 \| 2019 \| \| --- \| --- \| --- \| --- \| --- \| \| –93 \| –99 \| –06 \| –16 \| –21 \| \|  \|  \|  \|  \|  \| \|  \|  \|  \|  \|  \| \| 84 \| 70 \| 61 \| 19.2 \| 7.5 \| \| [83, 85] \| [69, 72] \| [59, 63] \| [18.8, 19.7] \| [7.2, 7.8] \| \|  \|  \|  \|  \|  \| \|  \|  \|  \|  \|  \| \| 89 \| 62 \| 28 \| 3 \| 1.3 \| \| [85, 92] \| [55, 68] \| [23, 34] \| [2, 5] \| [0.9, 1.9] \| \| 84 \| 66 \| 70 \| 42 \| 16 \| \| [79, 89] \| [57, 74] \| [62, 77] \| [39, 45] \| [14, 19] \| \| 92 \| 85 \| 76 \| 20 \| 7 \| \| [89, 94] \| [81, 89] \| [70, 81] \| [18, 22] \| [6, 8] \| \| 95 \| 92 \| 86 \| 39 \| 21 \| \| [93, 97] \| [90, 94] \| [83, 89] \| [38, 40] \| [19, 22] \| \| 94 \| 83 \| 74 \| 14 \| 4 \| \| [88, 97] \| [75, 89] \| [69, 79] \| [12, 15] \| [3, 5] \| \| 24 \| 13 \| 6 \| 0.5 \| 0.3 \| \| [18, 30] \| [9, 19] \| [4, 10] \| [0.1, 2] \| [0.0, 1.9] \| \| 70 \| 55 \| 43 \| 6 \| 2.0 \| \| [65, 75] \| [49, 60] \| [37, 48] \| [5, 7] \| [1.6, 2.5] \| \| 91 \| 78 \| 64 \| 12 \| 3.3 \| \| [89, 93] \| [74, 82] \| [58, 70] \| [10, 14] \| [2.6, 4.1] \| \| 89 \| 68 \| 46 \| 15 \| 3 \| \| [87, 91] \| [62, 73] \| [39, 53] \| [13, 18] \| [2, 4] \| \| 93 \| 87 \| 81 \| 29 \| 14 \| \| [85, 97] \| [82, 91] \| [76, 85] \| [27, 31] \| [12, 15] \| \| 77 \| 54 \| 32 \| 4 \| 1.5 \| \| [72, 81] \| [48, 60] \| [27, 37] \| [3, 5] \| [1.2, 1.9] \| \| 27 \| 8 \| 0.8 \| 0.14 \| 0.5 \| \| [22, 34] \| [5, 12] \| [0.4, 2] \| [0.06, 0.35] \| [0.3, 0.9] \| \| 92 \| 82 \| 73 \| 16 \| 5.1 \| \| [89, 94] \| [79, 85] \| [69, 77] \| [15, 17] \| [4.5, 5.7] \| \| 63 \| 47 \| 24 \| 4 \| 3 \| \| [57, 68] \| [42, 52] \| [20, 28] \| [3, 5] \| [2, 4] \| \| 81 \| 66 \| 51 \| 27 \| 15 \| \| [73, 87] \| [55, 75] \| [45, 57] \| [24, 30] \| [12, 18] \| \| 71 \| 81 \| 65 \| 34 \| 12 \| \| [62, 79] \| [73, 88] \| [57, 73] \| [30, 38] \| [10, 14] \| \| 40 \| 27 \| 16 \| 11 \| 7 \| \| [32, 49] \| [19, 36] \| [10, 24] \| [8, 14] \| [5, 9] \| \| 98.6 \| 87 \| 89 \| 59 \| 44 \| \| [96.7, 99.4] \| [81, 92] \| [86, 91] \| [56, 63] \| [40, 48] \| \| 94 \| 78 \| 55 \| 5.4 \| 1.4 \| \| [92, 95] \| [74, 81] \| [50, 60] \| [4.7, 6.3] \| [1.1, 1.9] \| \| 90 \| 71 \| 50 \| 6 \| 4 \| \| [87, 92] \| [66, 75] \| [44, 55] \| [4, 7] \| [3, 5] \| \| 96 \| 88 \| 74 \| 16 \| 4 \| \| [95, 97] \| [85, 90] \| [70, 79] \| [15, 17] \| [3, 5] \| \|  \| 70 \| 45 \| 2 \| 0.9 \| \|  \| [63, 77] \| [38, 52] \| [1, 3] \| [0.4, 2] \| \| 60 \| 23 \| 8 \| 0.9 \| 0.6 \| \| [54, 65] \| [19, 28] \| [6, 10] \| [0.7, 1.3] \| [0.3, 0.9] \| \| 78 \| 48 \| 28 \| 2.3 \| 1.5 \| \| [70, 84] \| [39, 57] \| [23, 34] \| [1.7, 3.2] \| [1.1, 2.0] \| \| 81 \| 62 \| 53 \| 16 \| 7 \| \| [74, 86] \| [53, 70] \| [44, 61] \| [13, 20] \| [6, 9] \| \| 96.5 \| 92 \| 87 \| 41.5 \| 12 \| \| [95.5, 97.3] \| [90, 93] \| [85, 89] \| [40.5, 42.4] \| [11, 13] \| \| 87 \| 86 \| 68 \| 30 \| 11 \| \| [77, 93] \| [77, 91] \| [62, 74] \| [27, 32] \| [9, 13] \| \| 77 \| 61 \| 53 \| 12 \| 2.3 \| \| [72, 80] \| [55, 66] \| [47, 59] \| [10, 14] \| [1.9, 2.8] \| \|  \|  \|  \|  \|  \| \|  \|  \|  \|  \|  \| \|  \|  \|  \| 1.3 \| 0.7 \| \|  \|  \|  \| [0.6, 2.6] \| [0.2, 2] \| \|  \|  \|  \| 4 \| 2 \| \|  \|  \|  \| [2, 9] \| [0.8, 7] \| \|  \|  \|  \| 9 \| 2 \| \|  \|  \|  \| [6, 14] \| [1, 4] \| \| 92 \| 84 \| 70 \| 17 \| 6 \| \| [89, 94] \| [80, 87] \| [65, 75] \| [16, 19] \| [5, 7] \| \|  \|  \|  \| 13 \| 4 \| \|  \|  \|  \| [10, 18] \| [3, 8] \| \|  \|  \|  \| 0.4 \| 0.4 \| \|  \|  \|  \| [0.1, 2.4] \| [0.1, 2.4] \| \| 66 \| 43 \| 40 \| 10 \| 3.3 \| \| [61, 70] \| [37, 50] \| [33, 46] \| [7, 15] \| [2.6, 4.3] \| \|  \|  \|  \| 0.4 \| 0.8 \| \|  \|  \|  \| [0.1, 1.3] \| [0.1, 5] \| |

| Low birth weight (selection corrected) quintiles: | Not weighed at birth quintiles: |
| --- | --- |
| \| 3.46 – 15.49 \| 15.61 – 18.36 \| 18.38 – 22.32 \| 22.44 – 28.54 \| 29.62 – 54.81 \| \| --- \| --- \| --- \| --- \| --- \| | \| 0.14 – 3.84 \| 3.96 – 15.66 \| 15.90 – 51.34 \| 52.66 – 77.87 \| 77.91 – 98.58 \| \| --- \| --- \| --- \| --- \| --- \| |

Notes: 95% confidence intervals are shown in square brackets below estimates. The colors represent quintiles. Selection corrected results were obtained using Heckman selection models for each survey: in a first stage regression, whether a child was weighed at birth or not was predicted using wealth index z-scores (linear and squared), maternal education (dummy coded), place of birth (dummy coded into public facility, private facility, other facility, or no facility), state (dummy coded), and sex. Estimates are rounded to whole numbers (except numbers below 1 and when confidence intervals would overlap).

## Table S7: Percentage point (pp) change in prevalence across surveys: Selection models for low birth weight and percentage not weighed at birth

|  | Low birth weight (selection corrected) | Not weighed at birth |
| --- | --- | --- |
| \|  \| \| --- \| \|  \| \|  \| \|  \| \| India \| \|  \| \|  \| \| States: \| \| Andhra Pradesh \| \|  \| \| Arunachal Pradesh \| \|  \| \| Assam \| \|  \| \| Bihar \| \|  \| \| Chhattisgarh \| \|  \| \| Goa \| \|  \| \| Gujarat \| \|  \| \| Haryana \| \|  \| \| Himachal Pradesh \| \|  \| \| Jharkhand \| \|  \| \| Karnataka \| \|  \| \| Kerala \| \|  \| \| Madhya Pradesh \| \|  \| \| Maharashtra \| \|  \| \| Manipur \| \|  \| \| Meghalaya \| \|  \| \| Mizoram \| \|  \| \| Nagaland \| \|  \| \| Odisha \| \|  \| \| Punjab \| \|  \| \| Rajasthan \| \|  \| \| Sikkim \| \|  \| \| Tamil Nadu \| \|  \| \| Telangana \| \|  \| \| Tripura \| \|  \| \| Uttar Pradesh \| \|  \| \| Uttarakhand \| \|  \| \| West Bengal \| \|  \| \|  \| \| Union territories: \| \| Andaman & Nico- \| \| bar Islands \| \| Chandigarh \| \|  \| \| Dadra & Nagar Haveli \| \| & Daman & Diu \| \| Jammu & Kashmir \| \|  \| \| Ladakh \| \|  \| \| Lakshadweep \| \|  \| \| Nct of Delhi \| \|  \| \| Puducherry \| \|  \| | \| 1992/3– \| 1992/3– \| 1998/9– \| 2005/6– \| 2015/6– \| \| --- \| --- \| --- \| --- \| --- \| \| 2019/21 \| 1998/9 \| 2005/6 \| 2015/6 \| 2019/21 \| \|  \|  \|  \|  \|  \| \|  \|  \|  \|  \|  \| \| -14*** \| -1 \| -4* \| -9*** \| -0.2 \| \| [-19, -9] \| [-7, 5] \| [-8, 0.3] \| [-11, -7] \| [-3, 3] \| \|  \|  \|  \|  \|  \| \|  \|  \|  \|  \|  \| \| -17*** \| -14** \| 1.0 \| -3* \| -1 \| \| [-27, -7] \| [-25, -2] \| [-6, 8] \| [-7, 0.5] \| [-4, 1.0] \| \| -12** \| 5 \| -10** \| -7*** \| -0.7 \| \| [-22, -2] \| [-6, 17] \| [-19, -0.8] \| [-12, -2] \| [-6, 4] \| \| -11** \| 12* \| -14*** \| -8*** \| 0.1 \| \| [-20, -1] \| [-0.9, 24] \| [-24, -4] \| [-13, -3] \| [-3, 3] \| \| -8 \| -2 \| 11** \| -19*** \| 2 \| \| [-18, 3] \| [-14, 10] \| [0.7, 21] \| [-25, -12] \| [-4, 8] \| \| -33*** \| -17 \| -10 \| -9*** \| 3*** \| \| [-51, -16] \| [-39, 4] \| [-24, 4] \| [-14, -4] \| [0.8, 6] \| \| -12*** \| 0.2 \| -4 \| -0.8 \| -8*** \| \| [-18, -7] \| [-7, 8] \| [-11, 3] \| [-6, 5] \| [-14, -2] \| \| -6*** \| -1 \| 1 \| -6*** \| -0.6 \| \| [-11, -2] \| [-8, 5] \| [-4, 7] \| [-9, -2] \| [-2, 1] \| \| -12** \| -3 \| 8** \| -17*** \| -0.1 \| \| [-21, -2] \| [-15, 8] \| [0.1, 17] \| [-22, -11] \| [-2, 2] \| \| -16*** \| 6 \| -10** \| -8*** \| -4** \| \| [-25, -8] \| [-5, 17] \| [-18, -2] \| [-13, -3] \| [-7, -0.7] \| \| -32*** \| -4 \| -19** \| -10*** \| 0.8 \| \| [-43, -21] \| [-22, 15] \| [-35, -3] \| [-15, -5] \| [-4, 6] \| \| -9*** \| -4 \| -0.1 \| -4*** \| -1 \| \| [-15, -3] \| [-11, 3] \| [-5, 5] \| [-6, -0.9] \| [-3, 0.7] \| \| -3* \| -1 \| -2 \| -0.8 \| 0.8 \| \| [-6, 0.0] \| [-5, 3] \| [-6, 2] \| [-4, 3] \| [-2, 3] \| \| -20*** \| -2 \| -11** \| -6*** \| -1 \| \| [-29, -12] \| [-13, 10] \| [-20, -2] \| [-10, -2] \| [-4, 0.9] \| \| -15*** \| -6** \| -5** \| -4*** \| 0.4 \| \| [-20, -10] \| [-12, -0.4] \| [-10, -0.0] \| [-7, -1] \| [-2, 3] \| \| -23*** \| -17*** \| 3 \| -7*** \| -2 \| \| [-34, -12] \| [-28, -6] \| [-2, 8] \| [-10, -4] \| [-7, 2] \| \| -11** \| -3 \| 3 \| -9*** \| -0.8 \| \| [-19, -2] \| [-13, 6] \| [-5, 11] \| [-14, -5] \| [-6, 5] \| \| -3 \| 2 \| -0.9 \| -3* \| -2 \| \| [-7, 0.8] \| [-2, 7] \| [-5, 3] \| [-5, 0.1] \| [-5, 0.8] \| \| -14 \| -5 \| 4 \| -8*** \| -4 \| \| [-39, 12] \| [-30, 20] \| [-7, 16] \| [-13, -3] \| [-16, 8] \| \| -8* \| 0.2 \| -5 \| -2 \| -2* \| \| [-17, 0.6] \| [-11, 11] \| [-12, 3] \| [-6, 1] \| [-3, 0.1] \| \| -12** \| -6 \| 4 \| -14*** \| 5*** \| \| [-22, -2] \| [-18, 5] \| [-4, 12] \| [-19, -9] \| [2, 8] \| \| -37*** \| -19** \| -4 \| -11*** \| -4*** \| \| [-51, -24] \| [-34, -4] \| [-12, 5] \| [-17, -5] \| [-6, -2] \| \|  \|  \| -15*** \| -4** \| 1 \| \|  \|  \| [-24, -6] \| [-8, -0.2] \| [-4, 6] \| \| -8*** \| -7** \| -0.7 \| -1 \| 0.5 \| \| [-13, -4] \| [-12, -1] \| [-4, 3] \| [-4, 1] \| [-1, 2] \| \| -14*** \| -6 \| -0.3 \| -5** \| -2 \| \| [-22, -5] \| [-15, 3] \| [-6, 5] \| [-9, -1.0] \| [-5, 0.5] \| \| -11** \| -3 \| 2 \| -12*** \| 2 \| \| [-21, -2] \| [-16, 10] \| [-9, 13] \| [-18, -6] \| [-2, 6] \| \| -12** \| 12* \| -11** \| -12*** \| -0.8 \| \| [-23, -2] \| [-1, 25] \| [-22, -1] \| [-17, -7] \| [-5, 4] \| \| -29*** \| 2 \| -19** \| -5* \| -8*** \| \| [-43, -16] \| [-18, 23] \| [-36, -2] \| [-12, 0.7] \| [-12, -3] \| \| -9*** \| 0.8 \| -3 \| -9*** \| 2* \| \| [-14, -4] \| [-6, 8] \| [-9, 3] \| [-13, -5] \| [-0.0, 4] \| \|  \|  \|  \|  \|  \| \|  \|  \|  \|  \|  \| \|  \|  \|  \|  \| 1 \| \|  \|  \|  \|  \| [-5, 7] \| \|  \|  \|  \|  \| -6 \| \|  \|  \|  \|  \| [-16, 4] \| \|  \|  \|  \|  \| -0.9 \| \|  \|  \|  \|  \| [-7, 5] \| \| -28*** \| -4 \| -9 \| -12*** \| -4** \| \| [-39, -17] \| [-18, 11] \| [-20, 3] \| [-18, -6] \| [-7, -0.6] \| \|  \|  \|  \|  \| 2 \| \|  \|  \|  \|  \| [-3, 8] \| \|  \|  \|  \|  \| -9** \| \|  \|  \|  \|  \| [-16, -1] \| \| -10*** \| -2 \| 0.6 \| -4 \| -5* \| \| [-15, -4] \| [-9, 5] \| [-6, 8] \| [-10, 3] \| [-10, 0.7] \| \|  \|  \|  \|  \| -2 \| \|  \|  \|  \|  \| [-7, 3] \| | \| 1992/3– \| 1992/3– \| 1998/9– \| 2005/6– \| 2015/6– \| \| --- \| --- \| --- \| --- \| --- \| \| 2019/21 \| 1998/9 \| 2005/6 \| 2015/6 \| 2019/21 \| \|  \|  \|  \|  \|  \| \|  \|  \|  \|  \|  \| \| -77*** \| -14*** \| -9*** \| -42*** \| -11.7*** \| \| [-78, -76] \| [-16, -12] \| [-12, -7] \| [-44, -40] \| [-12.2, -11.2] \| \|  \|  \|  \|  \|  \| \|  \|  \|  \|  \|  \| \| -88*** \| -27*** \| -34*** \| -25*** \| -2*** \| \| [-91, -84] \| [-34, -20] \| [-42, -25] \| [-31, -20] \| [-3, -0.5] \| \| -68*** \| -18*** \| 4 \| -28*** \| -25*** \| \| [-74, -62] \| [-28, -9] \| [-7, 15] \| [-36, -20] \| [-29, -22] \| \| -85*** \| -7*** \| -9*** \| -56*** \| -13*** \| \| [-88, -82] \| [-11, -2] \| [-16, -3] \| [-62, -50] \| [-15, -11] \| \| -75*** \| -3*** \| -6*** \| -47*** \| -18*** \| \| [-77, -73] \| [-6, -1.0] \| [-9, -2] \| [-50, -44] \| [-20, -17] \| \| -90*** \| -11*** \| -9** \| -60*** \| -10*** \| \| [-94, -86] \| [-19, -3] \| [-17, -0.8] \| [-65, -55] \| [-11, -8] \| \| -23*** \| -11*** \| -6** \| -6*** \| -0.3 \| \| [-30, -17] \| [-19, -3] \| [-12, -0.8] \| [-9, -3] \| [-1, 0.6] \| \| -68*** \| -15*** \| -12*** \| -37*** \| -4*** \| \| [-73, -63] \| [-23, -8] \| [-20, -4] \| [-42, -31] \| [-5, -3] \| \| -88*** \| -14*** \| -14*** \| -52*** \| -9*** \| \| [-90, -86] \| [-18, -9] \| [-21, -6] \| [-58, -46] \| [-11, -7] \| \| -86*** \| -21*** \| -22*** \| -31*** \| -12*** \| \| [-88, -83] \| [-27, -16] \| [-30, -13] \| [-38, -23] \| [-15, -9] \| \| -79*** \| -6 \| -6* \| -52*** \| -16*** \| \| [-85, -74] \| [-13, 1] \| [-13, 0.1] \| [-57, -47] \| [-18, -13] \| \| -75*** \| -22*** \| -22*** \| -28*** \| -2*** \| \| [-79, -71] \| [-30, -15] \| [-30, -14] \| [-33, -23] \| [-3, -1] \| \| -27*** \| -20*** \| -7*** \| -0.6** \| 0.4** \| \| [-33, -21] \| [-26, -13] \| [-11, -3] \| [-1.2, -0.0] \| [0.1, 0.7] \| \| -86*** \| -9*** \| -9*** \| -58*** \| -10*** \| \| [-89, -84] \| [-13, -5] \| [-14, -4] \| [-62, -54] \| [-12, -9] \| \| -60*** \| -16*** \| -23*** \| -20*** \| -1** \| \| [-65, -54] \| [-23, -8] \| [-30, -17] \| [-24, -16] \| [-2, -0.1] \| \| -66*** \| -15** \| -14** \| -25*** \| -12*** \| \| [-73, -59] \| [-27, -3] \| [-26, -3] \| [-31, -18] \| [-16, -8] \| \| -59*** \| 10* \| -16*** \| -31*** \| -22*** \| \| [-68, -50] \| [-1, 21] \| [-27, -5] \| [-40, -22] \| [-26, -18] \| \| -34*** \| -13** \| -11* \| -5 \| -4** \| \| [-42, -25] \| [-26, -1] \| [-22, 0.1] \| [-13, 2] \| [-8, -0.4] \| \| -54*** \| -12*** \| 2 \| -29*** \| -15*** \| \| [-59, -50] \| [-17, -6] \| [-4, 8] \| [-33, -25] \| [-20, -10] \| \| -92*** \| -16*** \| -23*** \| -50*** \| -4*** \| \| [-94, -91] \| [-20, -12] \| [-29, -16] \| [-55, -45] \| [-5, -3] \| \| -86*** \| -19*** \| -21*** \| -44*** \| -2** \| \| [-89, -83] \| [-24, -14] \| [-28, -14] \| [-49, -38] \| [-4, -0.0] \| \| -92*** \| -9*** \| -13*** \| -59*** \| -12*** \| \| [-94, -91] \| [-11, -6] \| [-18, -8] \| [-63, -54] \| [-13, -10] \| \|  \|  \| -25*** \| -43*** \| -0.9 \| \|  \|  \| [-36, -15] \| [-50, -36] \| [-2, 0.3] \| \| -59*** \| -36*** \| -16*** \| -7*** \| -0.4* \| \| [-64, -54] \| [-43, -29] \| [-21, -11] \| [-9, -4] \| [-0.8, 0.1] \| \| -76*** \| -30*** \| -20*** \| -26*** \| -0.9* \| \| [-83, -69] \| [-41, -18] \| [-30, -9] \| [-31, -21] \| [-2, 0.0] \| \| -73*** \| -19*** \| -9 \| -36*** \| -9*** \| \| [-79, -67] \| [-29, -8] \| [-22, 3] \| [-45, -27] \| [-13, -5] \| \| -84*** \| -5*** \| -4*** \| -46*** \| -29*** \| \| [-86, -83] \| [-7, -3] \| [-7, -2] \| [-48, -44] \| [-31, -28] \| \| -76*** \| -1 \| -17*** \| -38*** \| -19*** \| \| [-84, -68] \| [-12, 9] \| [-27, -8] \| [-45, -32] \| [-22, -16] \| \| -74*** \| -15*** \| -8** \| -41*** \| -9*** \| \| [-78, -70] \| [-22, -8] \| [-16, -0.3] \| [-47, -35] \| [-11, -7] \| \|  \|  \|  \|  \|  \| \|  \|  \|  \|  \|  \| \|  \|  \|  \|  \| -0.6 \| \|  \|  \|  \|  \| [-2, 0.6] \| \|  \|  \|  \|  \| -1 \| \|  \|  \|  \|  \| [-6, 3] \| \|  \|  \|  \|  \| -7*** \| \|  \|  \|  \|  \| [-11, -3] \| \| -86*** \| -8*** \| -14*** \| -53*** \| -11*** \| \| [-88, -84] \| [-12, -4] \| [-20, -7] \| [-58, -48] \| [-14, -9] \| \|  \|  \|  \|  \| -9*** \| \|  \|  \|  \|  \| [-14, -4] \| \|  \|  \|  \|  \| -0.0 \| \|  \|  \|  \|  \| [-1.0, 1.0] \| \| -62*** \| -22*** \| -4 \| -29*** \| -7*** \| \| [-67, -58] \| [-30, -14] \| [-13, 5] \| [-37, -21] \| [-11, -3] \| \|  \|  \|  \|  \| 0.4 \| \|  \|  \|  \|  \| [-1, 2] \| |

| Low birth weight (selection corrected) quintiles: | Not weighed at birth quintiles: |
| --- | --- |
| \| -37.32 – -11.91 \| -11.76 – -6.55 \| -6.36 – -3.32 \| -3.29 – -0.34 \| -0.09 – 11.73 \| \| --- \| --- \| --- \| --- \| --- \| | \| -92.39 – -55.65 \| -54.35 – -24.61 \| -23.43 – -13.52 \| -13.51 – -7.04 \| -7.03 – 9.99 \| \| --- \| --- \| --- \| --- \| --- \| |

Notes: *p<0.1; **p<0.05; p<0.01. 95% confidence intervals are shown in square brackets below estimates. The colors represent quintiles. Selection corrected results were obtained using Heckman selection models for each survey: in a first stage regression, whether a child was weighed at birth or not was predicted using wealth index z-scores (linear and squared), maternal education (dummy coded), place of birth (dummy coded into public facility, private facility, other facility, or no facility), state (dummy coded), and sex. Estimates are rounded to whole numbers (except numbers below 1 and when confidence intervals would overlap).

## Figure S3. Summary distribution of state and union territory-level prevalence, 1993-2021: Selection models for low birth weight and percentage not weighed at birth

Notes: Percentiles 5 and 95 (line) and 25, 50, and 75 (box) are shown. Dots indicate state estimates. States were equally weighted for the median and percentiles. Selection corrected results were obtained using Heckman selection models for each survey: in a first stage regression, whether a child was weighed at birth or not was predicted using wealth index z-scores (linear and squared), maternal education (dummy coded), place of birth (dummy coded into public facility, private facility, other facility, or no facility), state (dummy coded), and sex.

## Figure S4. Relationship between prevalence (%) in 1993 and percentage points (pp) change for 1993–2021: Selection models for low birth weight and percentage not weighed at birth

Notes: State labels were randomly displaced by at most 10% on either axis for readability. Selection corrected results were obtained using Heckman selection models for each survey: in a first stage regression, whether a child was weighed at birth or not was predicted using wealth index z-scores (linear and squared), maternal education (dummy coded), place of birth (dummy coded into public facility, private facility, other facility, or no facility), state (dummy coded), and sex. Andhra Pradesh (AP), Arunachal Pradesh (AR), Assam (AS), Bihar (BR), Chhattisgarh (CG), Goa (GA), Gujarat (GJ), Haryana (HR), Himachal Pradesh (HP), Jammu & Kashmir (JK), Jharkhand (JH), Karnataka (KA), Kerala (KL), Madhya Pradesh (MP), Maharashtra (MH), Manipur (MN), Meghalaya (ML), Mizoram (MZ), Nagaland (NL), Nct of Delhi (DL), Odisha (OR), Punjab (PB), Rajasthan (RJ), Tamil Nadu (TN), Telangana (TL), Tripura (TR), Uttar Pradesh (UP), Uttarakhand (UK), West Bengal (WB).

## Table S8: Prevalence (%) by survey: Low birth weight after reweighting samples to match wave five

|  | LBW (reweighted to match '19–21) | LBW (original) |
| --- | --- | --- |
| \|  \| \| --- \| \|  \| \|  \| \|  \| \| India \| \|  \| \|  \| \| States: \| \| Andhra Pradesh \| \|  \| \| Arunachal Pradesh \| \|  \| \| Assam \| \|  \| \| Bihar \| \|  \| \| Chhattisgarh \| \|  \| \| Goa \| \|  \| \| Gujarat \| \|  \| \| Haryana \| \|  \| \| Himachal Pradesh \| \|  \| \| Jharkhand \| \|  \| \| Karnataka \| \|  \| \| Kerala \| \|  \| \| Madhya Pradesh \| \|  \| \| Maharashtra \| \|  \| \| Manipur \| \|  \| \| Meghalaya \| \|  \| \| Mizoram \| \|  \| \| Nagaland \| \|  \| \| Odisha \| \|  \| \| Punjab \| \|  \| \| Rajasthan \| \|  \| \| Sikkim \| \|  \| \| Tamil Nadu \| \|  \| \| Telangana \| \|  \| \| Tripura \| \|  \| \| Uttar Pradesh \| \|  \| \| Uttarakhand \| \|  \| \| West Bengal \| \|  \| \|  \| \| Union territories: \| \| Andaman & Nico- \| \| bar Islands \| \| Chandigarh \| \|  \| \| Dadra & Nagar Haveli \| \| & Daman & Diu \| \| Jammu & Kashmir \| \|  \| \| Ladakh \| \|  \| \| Lakshadweep \| \|  \| \| Nct of Delhi \| \|  \| \| Puducherry \| \|  \| | \| 1992 \| 1998 \| 2005 \| 2015 \| 2019 \| \| --- \| --- \| --- \| --- \| --- \| \| –93 \| –99 \| –06 \| –16 \| –21 \| \|  \|  \|  \|  \|  \| \|  \|  \|  \|  \|  \| \| 29 \| 30 \| 25 \| 18.5 \| 18.2 \| \| [25, 33] \| [27, 33] \| [23, 28] \| [18.2, 18.8] \| [18.0, 18.5] \| \|  \|  \|  \|  \|  \| \|  \|  \|  \|  \|  \| \| 41 \| 17 \| 20 \| 18 \| 16 \| \| [26, 57] \| [12, 23] \| [16, 24] \| [16, 20] \| [15, 18] \| \| 19 \| 38 \| 15 \| 12 \| 11 \| \| [10, 34] \| [26, 51] \| [10, 22] \| [10, 14] \| [9, 12] \| \| 19 \| 36 \| 19 \| 17 \| 16 \| \| [9, 36] \| [22, 54] \| [12, 28] \| [16, 18] \| [15, 17] \| \| 24 \| 20 \| 37 \| 14.4 \| 17 \| \| [10, 47] \| [9, 39] \| [26, 50] \| [13.7, 15.1] \| [16, 18] \| \| 47 \| 42 \| 18 \| 13 \| 16 \| \| [19, 77] \| [25, 60] \| [14, 23] \| [12, 14] \| [15, 17] \| \| 27 \| 25 \| 22 \| 22 \| 14 \| \| [22, 32] \| [19, 33] \| [19, 25] \| [17, 27] \| [10, 19] \| \| 23 \| 22 \| 23 \| 19 \| 19 \| \| [18, 29] \| [17, 28] \| [19, 28] \| [18, 21] \| [17, 20] \| \| 23 \| 25 \| 32 \| 21 \| 21 \| \| [16, 33] \| [18, 32] \| [27, 37] \| [19, 22] \| [19, 22] \| \| 27 \| 38 \| 27 \| 20 \| 16 \| \| [19, 37] \| [29, 48] \| [21, 34] \| [17, 22] \| [14, 18] \| \| 63 \| 50 \| 20 \| 15 \| 16 \| \| [46, 78] \| [33, 68] \| [14, 27] \| [14, 16] \| [15, 17] \| \| 21 \| 20 \| 20 \| 17 \| 16 \| \| [15, 28] \| [16, 25] \| [18, 22] \| [16, 18] \| [15, 17] \| \| 18 \| 16 \| 17 \| 16 \| 16 \| \| [15, 20] \| [13, 19] \| [14, 21] \| [15, 18] \| [15, 18] \| \| 26 \| 32 \| 23 \| 22 \| 20.5 \| \| [16, 39] \| [22, 44] \| [19, 28] \| [21, 23] \| [19.7, 21.4] \| \| 34 \| 29 \| 23 \| 19 \| 20 \| \| [29, 39] \| [24, 33] \| [21, 26] \| [18, 21] \| [19, 21] \| \| 32 \| 11 \| 15 \| 10 \| 7 \| \| [16, 54] \| [6, 22] \| [10, 22] \| [9, 11] \| [6, 9] \| \| 11 \| 10 \| 15 \| 13 \| 12 \| \| [5, 23] \| [4, 25] \| [7, 30] \| [11, 16] \| [10, 13] \| \| 8 \| 11 \| 9 \| 6 \| 4 \| \| [4, 17] \| [7, 18] \| [7, 12] \| [5, 8] \| [3, 5] \| \| 20 \| 15 \| 11 \| 9 \| 5 \| \| [3, 69] \| [3, 50] \| [6, 19] \| [7, 11] \| [4, 6] \| \| 30 \| 28 \| 22 \| 21 \| 19 \| \| [15, 50] \| [20, 38] \| [19, 26] \| [20, 22] \| [18, 20] \| \| 30 \| 27 \| 29 \| 18 \| 22 \| \| [21, 41] \| [19, 37] \| [24, 34] \| [17, 20] \| [21, 24] \| \| 34 \| 29 \| 29 \| 21 \| 18 \| \| [23, 48] \| [22, 36] \| [23, 35] \| [20, 22] \| [17, 19] \| \|  \| 16 \| 12 \| 9 \| 10 \| \|  \| [9, 26] \| [8, 17] \| [6, 13] \| [6, 16] \| \| 23 \| 17 \| 16 \| 16 \| 17 \| \| [18, 28] \| [14, 20] \| [14, 19] \| [15, 17] \| [16, 18] \| \| 33 \| 20 \| 20 \| 16 \| 14 \| \| [21, 48] \| [15, 25] \| [16, 24] \| [14, 19] \| [13, 15] \| \| 37 \| 33 \| 45 \| 17 \| 20 \| \| [21, 55] \| [14, 60] \| [31, 61] \| [15, 20] \| [18, 22] \| \| 29 \| 48 \| 27 \| 21 \| 20.2 \| \| [17, 45] \| [36, 59] \| [21, 33] \| [20, 22] \| [19.5, 20.8] \| \| 50 \| 43 \| 29 \| 25 \| 18 \| \| [28, 72] \| [26, 62] \| [21, 40] \| [23, 27] \| [16, 20] \| \| 31 \| 28 \| 25 \| 17 \| 19 \| \| [25, 38] \| [22, 34] \| [21, 30] \| [16, 18] \| [18, 20] \| \|  \|  \|  \|  \|  \| \|  \|  \|  \|  \|  \| \|  \|  \|  \| 17 \| 17 \| \|  \|  \|  \| [13, 21] \| [13, 22] \| \|  \|  \|  \| 21 \| 17 \| \|  \|  \|  \| [15, 28] \| [11, 24] \| \|  \|  \|  \| 24 \| 21 \| \|  \|  \|  \| [20, 30] \| [17, 25] \| \| 27 \| 40 \| 30 \| 14 \| 11 \| \| [17, 40] \| [24, 58] \| [21, 41] \| [13, 16] \| [10, 12] \| \|  \|  \|  \| 10 \| 12 \| \|  \|  \|  \| [6, 15] \| [8, 16] \| \|  \|  \|  \| 19 \| 10 \| \|  \|  \|  \| [13, 27] \| [6, 14] \| \| 29 \| 27 \| 30 \| 25 \| 22 \| \| [21, 38] \| [20, 36] \| [25, 36] \| [22, 29] \| [20, 24] \| \|  \|  \|  \| 16 \| 14 \| \|  \|  \|  \| [14, 19] \| [10, 19] \| | \| 1992 \| 1998 \| 2005 \| 2015 \| 2019 \| \| --- \| --- \| --- \| --- \| --- \| \| –93 \| –99 \| –06 \| –16 \| –21 \| \|  \|  \|  \|  \|  \| \|  \|  \|  \|  \|  \| \| 26 \| 23 \| 21.5 \| 18.2 \| 18.2 \| \| [24, 27] \| [21, 24] \| [20.7, 22.4] \| [17.9, 18.5] \| [18.0, 18.5] \| \|  \|  \|  \|  \|  \| \|  \|  \|  \|  \|  \| \| 28 \| 18 \| 19 \| 18 \| 16 \| \| [20, 37] \| [13, 24] \| [16, 23] \| [16, 19] \| [15, 18] \| \| 20 \| 25 \| 14 \| 11 \| 11 \| \| [13, 29] \| [18, 34] \| [10, 19] \| [9, 12] \| [9, 12] \| \| 22 \| 33 \| 19 \| 16 \| 16 \| \| [15, 31] \| [26, 42] \| [15, 24] \| [15, 17] \| [15, 17] \| \| 18 \| 15 \| 28 \| 14.4 \| 17 \| \| [12, 25] \| [10, 23] \| [22, 34] \| [13.7, 15.1] \| [16, 18] \| \| 42 \| 28 \| 17 \| 13 \| 16 \| \| [27, 60] \| [16, 45] \| [13, 22] \| [12, 14] \| [15, 17] \| \| 25 \| 25 \| 22 \| 22 \| 14 \| \| [21, 30] \| [19, 32] \| [19, 26] \| [18, 27] \| [10, 19] \| \| 21 \| 20 \| 22 \| 19 \| 19 \| \| [18, 25] \| [16, 24] \| [19, 25] \| [18, 20] \| [17, 20] \| \| 26 \| 24 \| 33 \| 20 \| 21 \| \| [18, 36] \| [19, 31] \| [28, 38] \| [19, 22] \| [19, 22] \| \| 28 \| 35 \| 25 \| 20 \| 16 \| \| [21, 36] \| [29, 42] \| [21, 29] \| [18, 22] \| [14, 18] \| \| 41 \| 37 \| 19 \| 14.5 \| 16 \| \| [32, 49] \| [24, 53] \| [15, 24] \| [13.6, 15.5] \| [15, 17] \| \| 21 \| 17 \| 19 \| 17 \| 16 \| \| [17, 27] \| [14, 22] \| [17, 21] \| [16, 19] \| [15, 17] \| \| 18 \| 18 \| 16 \| 15 \| 16 \| \| [16, 21] \| [15, 21] \| [13, 20] \| [14, 17] \| [15, 18] \| \| 35 \| 33 \| 23 \| 22 \| 20.5 \| \| [29, 41] \| [26, 41] \| [20, 27] \| [21, 23] \| [19.7, 21.4] \| \| 32 \| 26 \| 22 \| 20 \| 20 \| \| [28, 36] \| [23, 30] \| [20, 25] \| [18, 21] \| [19, 21] \| \| 27 \| 10 \| 13 \| 9 \| 7 \| \| [18, 37] \| [7, 15] \| [11, 16] \| [8, 10] \| [6, 9] \| \| 19 \| 15 \| 18 \| 12 \| 12 \| \| [14, 26] \| [10, 22] \| [14, 23] \| [11, 14] \| [10, 13] \| \| 6 \| 9 \| 8 \| 6 \| 4 \| \| [3, 10] \| [6, 13] \| [6, 10] \| [5, 8] \| [3, 5] \| \| 11 \| 8 \| 11 \| 8 \| 5 \| \| [1, 53] \| [2, 30] \| [7, 16] \| [6, 10] \| [4, 6] \| \| 23 \| 25 \| 21 \| 21 \| 19 \| \| [16, 32] \| [19, 32] \| [17, 24] \| [20, 22] \| [18, 20] \| \| 29 \| 24 \| 28 \| 17 \| 22 \| \| [21, 38] \| [18, 31] \| [23, 32] \| [16, 19] \| [21, 24] \| \| 48 \| 30 \| 27 \| 21 \| 18 \| \| [36, 59] \| [25, 36] \| [22, 33] \| [20, 22] \| [17, 19] \| \|  \| 24 \| 10 \| 8 \| 10 \| \|  \| [17, 33] \| [8, 14] \| [7, 11] \| [6, 16] \| \| 23 \| 17 \| 17 \| 16 \| 17 \| \| [19, 27] \| [15, 20] \| [15, 20] \| [15, 17] \| [16, 18] \| \| 24 \| 19 \| 19 \| 16 \| 14 \| \| [17, 32] \| [15, 24] \| [16, 23] \| [14, 18] \| [13, 15] \| \| 28 \| 25 \| 27 \| 17 \| 20 \| \| [20, 36] \| [16, 36] \| [22, 33] \| [15, 20] \| [18, 22] \| \| 25 \| 37 \| 25 \| 20.7 \| 20.2 \| \| [18, 35] \| [29, 45] \| [21, 29] \| [20.0, 21.4] \| [19.5, 20.8] \| \| 40 \| 41 \| 25 \| 25 \| 18 \| \| [28, 53] \| [26, 58] \| [20, 30] \| [22, 27] \| [16, 20] \| \| 25 \| 25 \| 23 \| 17 \| 19 \| \| [21, 29] \| [21, 30] \| [20, 26] \| [15, 18] \| [18, 20] \| \|  \|  \|  \|  \|  \| \|  \|  \|  \|  \|  \| \|  \|  \|  \| 16 \| 17 \| \|  \|  \|  \| [12, 21] \| [13, 22] \| \|  \|  \|  \| 22 \| 17 \| \|  \|  \|  \| [16, 30] \| [11, 24] \| \|  \|  \|  \| 22 \| 21 \| \|  \|  \|  \| [18, 27] \| [17, 25] \| \| 33 \| 28 \| 19 \| 14 \| 11 \| \| [24, 43] \| [20, 38] \| [14, 26] \| [13, 15] \| [10, 12] \| \|  \|  \|  \| 9 \| 12 \| \|  \|  \|  \| [6, 13] \| [8, 16] \| \|  \|  \|  \| 18 \| 10 \| \|  \|  \|  \| [13, 26] \| [6, 14] \| \| 27 \| 26 \| 26 \| 27 \| 22 \| \| [24, 31] \| [22, 31] \| [22, 31] \| [22, 32] \| [20, 24] \| \|  \|  \|  \| 16 \| 14 \| \|  \|  \|  \| [13, 19] \| [10, 19] \| |

| \| LBW (reweighted to match '19–21) quintiles: \| LBW (original) quintiles: \| \| --- \| --- \| \| \| 4.02 – 14.61 \| 14.67 – 17.71 \| 17.85 – 20.84 \| 20.88 – 28.53 \| 28.60 – 63.31 \| \| --- \| --- \| --- \| --- \| --- \| \| \| 4.02 – 14.14 \| 14.40 – 17.65 \| 17.67 – 20.76 \| 20.84 – 25.31 \| 25.36 – 47.66 \| \| --- \| --- \| --- \| --- \| --- \| \| |  |
| --- | --- | --- | --- | --- | --- | --- | --- | --- | --- | --- | --- | --- | --- | --- | --- |
|  |  |

Notes: 95% confidence intervals are shown in square brackets below estimates. The colors represent quintiles. 'Reweighted' refers to results obtained after rescaling sample weights such that the distribution of demographic and socioeconomic characteristics is the same as the distribution among children with a valid birth weight measure in the latest survey (2019–21). Estimates are rounded to whole numbers (except numbers below 1 and when confidence intervals would overlap).

## Table S9: Percentage point (pp) change in prevalence across surveys: Low birth weight after reweighting samples to match wave five

|  | LBW (reweighted to match '19–21) | LBW (original) |
| --- | --- | --- |
| \|  \| \| --- \| \|  \| \|  \| \|  \| \| India \| \|  \| \|  \| \| States: \| \| Andhra Pradesh \| \|  \| \| Arunachal Pradesh \| \|  \| \| Assam \| \|  \| \| Bihar \| \|  \| \| Chhattisgarh \| \|  \| \| Goa \| \|  \| \| Gujarat \| \|  \| \| Haryana \| \|  \| \| Himachal Pradesh \| \|  \| \| Jharkhand \| \|  \| \| Karnataka \| \|  \| \| Kerala \| \|  \| \| Madhya Pradesh \| \|  \| \| Maharashtra \| \|  \| \| Manipur \| \|  \| \| Meghalaya \| \|  \| \| Mizoram \| \|  \| \| Nagaland \| \|  \| \| Odisha \| \|  \| \| Punjab \| \|  \| \| Rajasthan \| \|  \| \| Sikkim \| \|  \| \| Tamil Nadu \| \|  \| \| Telangana \| \|  \| \| Tripura \| \|  \| \| Uttar Pradesh \| \|  \| \| Uttarakhand \| \|  \| \| West Bengal \| \|  \| \|  \| \| Union territories: \| \| Andaman & Nico- \| \| bar Islands \| \| Chandigarh \| \|  \| \| Dadra & Nagar Haveli \| \| & Daman & Diu \| \| Jammu & Kashmir \| \|  \| \| Ladakh \| \|  \| \| Lakshadweep \| \|  \| \| Nct of Delhi \| \|  \| \| Puducherry \| \|  \| | \| 1992/3– \| 1992/3– \| 1998/9– \| 2005/6– \| 2015/6– \| \| --- \| --- \| --- \| --- \| --- \| \| 2019/21 \| 1998/9 \| 2005/6 \| 2015/6 \| 2019/21 \| \|  \|  \|  \|  \|  \| \|  \|  \|  \|  \|  \| \| -10*** \| 1 \| -5** \| -7*** \| -0.3 \| \| [-14, -6] \| [-4, 7] \| [-9, -0.6] \| [-9, -5] \| [-0.6, 0.1] \| \|  \|  \|  \|  \|  \| \|  \|  \|  \|  \|  \| \| -24*** \| -24*** \| 3 \| -2 \| -2 \| \| [-40, -8] \| [-40, -7] \| [-4, 9] \| [-6, 2] \| [-4, 0.5] \| \| -9 \| 18** \| -23*** \| -3 \| -1 \| \| [-21, 4] \| [0.7, 36] \| [-37, -9] \| [-9, 4] \| [-4, 1] \| \| -3 \| 18 \| -18* \| -2 \| -0.9 \| \| [-16, 11] \| [-4, 39] \| [-36, 0.5] \| [-9, 6] \| [-3, 0.7] \| \| -7 \| -3 \| 17* \| -23*** \| 2*** \| \| [-26, 12] \| [-28, 21] \| [-2, 36] \| [-35, -11] \| [1, 3] \| \| -32* \| -6 \| -24** \| -5** \| 3*** \| \| [-65, 2] \| [-44, 32] \| [-43, -5] \| [-10, -0.2] \| [1, 5] \| \| -13*** \| -2 \| -3 \| -0.2 \| -8** \| \| [-19, -6] \| [-10, 7] \| [-11, 5] \| [-6, 6] \| [-14, -1] \| \| -5 \| -1 \| 1 \| -4* \| -0.8 \| \| [-11, 1] \| [-9, 7] \| [-6, 8] \| [-8, 0.5] \| [-2, 0.8] \| \| -3 \| 1 \| 7 \| -11*** \| -0.1 \| \| [-12, 6] \| [-10, 13] \| [-2, 15] \| [-16, -6] \| [-2, 2] \| \| -11** \| 11 \| -11* \| -8** \| -4** \| \| [-21, -2] \| [-3, 24] \| [-23, 0.8] \| [-14, -0.8] \| [-7, -0.7] \| \| -48*** \| -13 \| -30*** \| -5 \| 1.0 \| \| [-64, -32] \| [-38, 12] \| [-50, -11] \| [-12, 1] \| [-0.4, 2] \| \| -5 \| -0.2 \| -0.5 \| -3* \| -1 \| \| [-11, 2] \| [-8, 8] \| [-6, 5] \| [-5, 0.1] \| [-3, 0.6] \| \| -1 \| -2 \| 0.8 \| -0.7 \| -0.0 \| \| [-4, 2] \| [-5, 2] \| [-4, 5] \| [-5, 3] \| [-3, 3] \| \| -5 \| 6 \| -9 \| -1.0 \| -2*** \| \| [-17, 6] \| [-10, 22] \| [-21, 3] \| [-6, 4] \| [-3, -0.6] \| \| -14*** \| -5 \| -5* \| -4** \| 0.6 \| \| [-19, -9] \| [-12, 2] \| [-10, 0.2] \| [-7, -0.8] \| [-1, 2] \| \| -25** \| -21* \| 4 \| -5* \| -3*** \| \| [-45, -5] \| [-42, 0.8] \| [-6, 14] \| [-11, 0.5] \| [-5, -0.8] \| \| 0.7 \| -0.9 \| 5 \| -2 \| -1 \| \| [-8, 9] \| [-14, 12] \| [-10, 20] \| [-14, 9] \| [-4, 2] \| \| -4 \| 3 \| -2 \| -3* \| -2** \| \| [-11, 2] \| [-5, 11] \| [-8, 3] \| [-6, 0.4] \| [-4, -0.3] \| \| -15 \| -5 \| -4 \| -2 \| -4*** \| \| [-49, 20] \| [-46, 37] \| [-28, 19] \| [-9, 5] \| [-7, -1] \| \| -11 \| -2 \| -6 \| -2 \| -1* \| \| [-29, 7] \| [-22, 18] \| [-15, 4] \| [-5, 2] \| [-3, 0.1] \| \| -8 \| -3 \| 2 \| -11*** \| 4*** \| \| [-18, 2] \| [-17, 11] \| [-9, 12] \| [-16, -6] \| [2, 7] \| \| -17*** \| -6 \| 0.1 \| -7** \| -4*** \| \| [-29, -4] \| [-20, 9] \| [-9, 10] \| [-14, -1] \| [-5, -2] \| \|  \|  \| -4 \| -2 \| 0.6 \| \|  \|  \| [-14, 5] \| [-8, 3] \| [-5, 7] \| \| -6** \| -6** \| -0.5 \| -0.0 \| 0.8 \| \| [-11, -0.7] \| [-12, -0.3] \| [-4, 3] \| [-3, 2] \| [-0.9, 2] \| \| -19*** \| -14* \| 0.2 \| -4 \| -2* \| \| [-33, -5] \| [-28, 1.0] \| [-6, 6] \| [-8, 0.8] \| [-5, 0.3] \| \| -17* \| -4 \| 12 \| -28*** \| 2 \| \| [-35, 0.9] \| [-34, 27] \| [-17, 42] \| [-44, -12] \| [-1, 6] \| \| -9 \| 19** \| -21*** \| -6* \| -0.7 \| \| [-23, 6] \| [0.5, 37] \| [-34, -8] \| [-12, 0.4] \| [-2, 0.3] \| \| -32*** \| -7 \| -14 \| -4 \| -7*** \| \| [-56, -8] \| [-37, 24] \| [-35, 7] \| [-14, 6] \| [-11, -4] \| \| -12*** \| -4 \| -3 \| -8*** \| 2** \| \| [-19, -6] \| [-13, 5] \| [-10, 5] \| [-13, -4] \| [0.1, 4] \| \|  \|  \|  \|  \|  \| \|  \|  \|  \|  \|  \| \|  \|  \|  \|  \| 0.8 \| \|  \|  \|  \|  \| [-5, 7] \| \|  \|  \|  \|  \| -4 \| \|  \|  \|  \|  \| [-13, 6] \| \|  \|  \|  \|  \| -4 \| \|  \|  \|  \|  \| [-10, 3] \| \| -16*** \| 14 \| -10 \| -16*** \| -4*** \| \| [-28, -4] \| [-8, 35] \| [-30, 10] \| [-26, -6] \| [-5, -2] \| \|  \|  \|  \|  \| 2 \| \|  \|  \|  \|  \| [-4, 7] \| \|  \|  \|  \|  \| -9** \| \|  \|  \|  \|  \| [-17, -1] \| \| -6 \| -1 \| 3 \| -5 \| -3 \| \| [-15, 2] \| [-13, 11] \| [-7, 13] \| [-12, 2] \| [-7, 1] \| \|  \|  \|  \|  \| -3 \| \|  \|  \|  \|  \| [-8, 2] \| | \| 1992/3– \| 1992/3– \| 1998/9– \| 2005/6– \| 2015/6– \| \| --- \| --- \| --- \| --- \| --- \| \| 2019/21 \| 1998/9 \| 2005/6 \| 2015/6 \| 2019/21 \| \|  \|  \|  \|  \|  \| \|  \|  \|  \|  \|  \| \| -8*** \| -3*** \| -1 \| -3*** \| 0.0 \| \| [-9, -6] \| [-5, -1] \| [-3, 0.4] \| [-4, -2] \| [-0.4, 0.4] \| \|  \|  \|  \|  \|  \| \|  \|  \|  \|  \|  \| \| -12*** \| -10* \| 2 \| -2 \| -1 \| \| [-20, -3] \| [-20, 0.2] \| [-5, 8] \| [-6, 2] \| [-4, 0.9] \| \| -9** \| 5 \| -11** \| -3 \| -0.1 \| \| [-18, -0.8] \| [-6, 17] \| [-20, -2] \| [-8, 1] \| [-2, 2] \| \| -6 \| 11* \| -14*** \| -4 \| 0.4 \| \| [-14, 2] \| [-0.0, 23] \| [-24, -4] \| [-8, 1] \| [-1, 2] \| \| -0.9 \| -2 \| 12*** \| -13*** \| 2*** \| \| [-8, 6] \| [-12, 7] \| [3, 22] \| [-19, -7] \| [1, 3] \| \| -26*** \| -15 \| -10 \| -5** \| 3*** \| \| [-44, -9] \| [-37, 8] \| [-26, 5] \| [-9, -0.3] \| [2, 5] \| \| -11*** \| -0.1 \| -3 \| 0.0 \| -8*** \| \| [-17, -6] \| [-8, 8] \| [-10, 4] \| [-6, 6] \| [-14, -2] \| \| -3 \| -1 \| 2 \| -3* \| -0.5 \| \| [-6, 0.8] \| [-7, 4] \| [-3, 7] \| [-6, 0.5] \| [-2, 1] \| \| -5 \| -2 \| 8** \| -12*** \| 0.1 \| \| [-14, 3] \| [-12, 9] \| [0.4, 16] \| [-17, -7] \| [-2, 2] \| \| -12*** \| 8 \| -10** \| -5** \| -4** \| \| [-20, -4] \| [-3, 18] \| [-18, -2] \| [-10, -0.6] \| [-7, -0.9] \| \| -25*** \| -3 \| -18** \| -5* \| 1* \| \| [-34, -16] \| [-20, 14] \| [-34, -3] \| [-9, 0.1] \| [-0.2, 2] \| \| -5** \| -4 \| 1 \| -2 \| -1 \| \| [-11, -0.4] \| [-10, 2] \| [-3, 6] \| [-4, 1] \| [-3, 0.6] \| \| -2 \| -0.6 \| -1 \| -0.6 \| 0.8 \| \| [-5, 1] \| [-4, 3] \| [-6, 3] \| [-4, 3] \| [-2, 3] \| \| -14*** \| -1 \| -10** \| -2 \| -1** \| \| [-21, -8] \| [-11, 8] \| [-18, -2] \| [-5, 2] \| [-2, -0.2] \| \| -12*** \| -6** \| -4* \| -3* \| 0.5 \| \| [-16, -8] \| [-12, -1] \| [-8, 0.5] \| [-5, 0.2] \| [-1, 2] \| \| -19*** \| -16*** \| 3 \| -4*** \| -2** \| \| [-29, -10] \| [-27, -6] \| [-2, 8] \| [-7, -1] \| [-4, -0.2] \| \| -7** \| -4 \| 3 \| -6** \| -0.5 \| \| [-14, -1] \| [-13, 4] \| [-4, 11] \| [-10, -1] \| [-3, 2] \| \| -2 \| 3 \| -1 \| -2 \| -2** \| \| [-5, 2] \| [-2, 7] \| [-5, 3] \| [-4, 1.0] \| [-4, -0.1] \| \| -6 \| -3 \| 2 \| -3 \| -3*** \| \| [-28, 15] \| [-27, 22] \| [-10, 15] \| [-8, 1] \| [-5, -0.9] \| \| -4 \| 2 \| -4 \| 0.2 \| -2** \| \| [-12, 4] \| [-8, 12] \| [-12, 3] \| [-3, 4] \| [-3, -0.1] \| \| -6 \| -5 \| 4 \| -11*** \| 5*** \| \| [-15, 3] \| [-16, 6] \| [-4, 12] \| [-15, -6] \| [3, 7] \| \| -30*** \| -17** \| -3 \| -6** \| -4*** \| \| [-42, -18] \| [-30, -4] \| [-11, 5] \| [-12, -0.5] \| [-5, -2] \| \|  \|  \| -14*** \| -2 \| 1 \| \|  \|  \| [-23, -5] \| [-5, 2] \| [-4, 7] \| \| -6*** \| -5** \| -0.0 \| -0.8 \| 0.6 \| \| [-10, -2] \| [-10, -0.6] \| [-4, 4] \| [-3, 2] \| [-1, 2] \| \| -10** \| -5 \| 0.6 \| -3* \| -2 \| \| [-17, -2] \| [-14, 4] \| [-5, 6] \| [-8, 0.6] \| [-5, 0.6] \| \| -8* \| -3 \| 2 \| -10*** \| 2 \| \| [-16, 0.3] \| [-15, 10] \| [-9, 13] \| [-15, -4] \| [-1, 6] \| \| -5 \| 11* \| -12** \| -4** \| -0.5 \| \| [-14, 3] \| [-0.4, 23] \| [-21, -2] \| [-9, -0.3] \| [-1.5, 0.4] \| \| -22*** \| 1 \| -16* \| 0.1 \| -7*** \| \| [-35, -9] \| [-20, 22] \| [-34, 2] \| [-6, 6] \| [-10, -4] \| \| -6** \| 0.8 \| -3 \| -6*** \| 2** \| \| [-10, -1] \| [-5, 7] \| [-8, 3] \| [-10, -3] \| [0.4, 4] \| \|  \|  \|  \|  \|  \| \|  \|  \|  \|  \|  \| \|  \|  \|  \|  \| 1 \| \|  \|  \|  \|  \| [-5, 7] \| \|  \|  \|  \|  \| -5 \| \|  \|  \|  \|  \| [-15, 4] \| \|  \|  \|  \|  \| -0.8 \| \|  \|  \|  \|  \| [-7, 5] \| \| -22*** \| -4 \| -9 \| -5* \| -3*** \| \| [-32, -12] \| [-18, 9] \| [-20, 2] \| [-12, 1.0] \| [-5, -2] \| \|  \|  \|  \|  \| 2 \| \|  \|  \|  \|  \| [-3, 8] \| \|  \|  \|  \|  \| -9** \| \|  \|  \|  \|  \| [-16, -1] \| \| -5** \| -1 \| 0.3 \| 0.1 \| -4 \| \| [-10, -1.0] \| [-7, 5] \| [-6, 7] \| [-7, 7] \| [-10, 1] \| \|  \|  \|  \|  \| -2 \| \|  \|  \|  \|  \| [-7, 3] \| |

| LBW (reweighted to match '19–21) quintiles: | LBW (original) quintiles: |
| --- | --- |
| \| -47.68 – -10.86 \| -10.68 – -4.68 \| -4.65 – -2.46 \| -2.45 – -0.03 \| -0.02 – 18.91 \| \| --- \| --- \| --- \| --- \| --- \| | \| -29.95 – -9.16 \| -8.76 – -4.35 \| -4.18 – -1.84 \| -1.80 – 0.12 \| 0.21 – 12.27 \| \| --- \| --- \| --- \| --- \| --- \| |

Notes: *p<0.1; **p<0.05; p<0.01. 95% confidence intervals are shown in square brackets below estimates. The colors represent quintiles. 'Reweighted' refers to results obtained after rescaling sample weights such that the distribution of demographic and socioeconomic characteristics is the same as the distribution among children with a valid birth weight measure in the latest survey (2019–21). Estimates are rounded to whole numbers (except numbers below 1 and when confidence intervals would overlap).

## Figure S5. Summary distribution of state and union territory-level prevalence, 1993-2021: Low birth weight after reweighting samples to match wave five

Notes: Percentiles 5 and 95 (line) and 25, 50, and 75 (box) are shown. Dots indicate state estimates. States were equally weighted for the median and percentiles. 'Reweighted' refers to results obtained after rescaling sample weights such that the distribution of demographic and socioeconomic characteristics is the same as the distribution among children with a valid birth weight measure in the latest survey (2019–21).

## Figure S6. Relationship between prevalence (%) in 1993 and percentage points (pp) change for 1993–2021: Low birth weight after reweighting samples to match wave five

Notes: State labels were randomly displaced by at most 10% on either axis for readability. 'Reweighted' refers to results obtained after rescaling sample weights such that the distribution of demographic and socioeconomic characteristics is the same as the distribution among children with a valid birth weight measure in the latest survey (2019–21). Andhra Pradesh (AP), Arunachal Pradesh (AR), Assam (AS), Bihar (BR), Chhattisgarh (CG), Goa (GA), Gujarat (GJ), Haryana (HR), Himachal Pradesh (HP), Jammu & Kashmir (JK), Jharkhand (JH), Karnataka (KA), Kerala (KL), Madhya Pradesh (MP), Maharashtra (MH), Manipur (MN), Meghalaya (ML), Mizoram (MZ), Nagaland (NL), Nct of Delhi (DL), Odisha (OR), Punjab (PB), Rajasthan (RJ), Tamil Nadu (TN), Telangana (TL), Tripura (TR), Uttar Pradesh (UP), Uttarakhand (UK), West Bengal (WB).

## Table S10: Estimated headcount and percentage share of low birth weight and small birth size across states and union territories in 2021

| Low birth weight | Small at birth |
| --- | --- |
| \|  \| Cumulative \| Births \| Percentage \| \| --- \| --- \| --- \| --- \| \|  \| percentage \| ('000) \| distribution \| \|  \|  \|  \|  \| \| India \|  \| 4,221 \| 100.00 \| \| Uttar Pradesh \| 20.33 \| 858 \| 20.33 \| \| Bihar \| 30.51 \| 430 \| 10.18 \| \| Maharashtra \| 39.95 \| 399 \| 9.45 \| \| West Bengal \| 47.48 \| 318 \| 7.53 \| \| Madhya Pradesh \| 54.43 \| 293 \| 6.95 \| \| Rajasthan \| 60.94 \| 275 \| 6.51 \| \| Tamil Nadu \| 65.72 \| 202 \| 4.78 \| \| Gujarat \| 70.29 \| 193 \| 4.57 \| \| Karnataka \| 74.43 \| 175 \| 4.14 \| \| Odisha \| 77.95 \| 149 \| 3.52 \| \| Andhra Pradesh \| 81.00 \| 129 \| 3.06 \| \| Jharkhand \| 83.43 \| 103 \| 2.43 \| \| Assam \| 85.71 \| 96 \| 2.28 \| \| Haryana \| 87.95 \| 95 \| 2.24 \| \| Punjab \| 90.19 \| 94 \| 2.24 \| \| Chhattisgarh \| 92.21 \| 85 \| 2.02 \| \| Kerala \| 94.12 \| 81 \| 1.92 \| \| Telangana \| 95.95 \| 77 \| 1.83 \| \| Nct of Delhi \| 97.55 \| 68 \| 1.60 \| \| Uttarakhand \| 98.29 \| 31 \| 0.73 \| \| Jammu & Kashmir \| 98.70 \| 18 \| 0.42 \| \| Himachal Pradesh \| 99.08 \| 16 \| 0.38 \| \| Tripura \| 99.36 \| 12 \| 0.28 \| \| Meghalaya \| 99.58 \| 9 \| 0.22 \| \| Goa \| 99.66 \| 3 \| 0.07 \| \| Manipur \| 99.73 \| 3 \| 0.07 \| \| Chandigarh \| 99.79 \| 3 \| 0.06 \| \| Puducherry \| 99.84 \| 2 \| 0.05 \| \| Dadra & Nagar Haveli & Daman & Diu \| 99.89 \| 2 \| 0.05 \| \| Arunachal Pradesh \| 99.93 \| 2 \| 0.04 \| \| Andaman & Nicobar Islands \| 99.94 \| 1 \| 0.02 \| \| Mizoram \| 99.96 \| 1 \| 0.02 \| \| Sikkim \| 99.98 \| 1 \| 0.02 \| \| Nagaland \| 99.99 \| 1 \| 0.01 \| \| Ladakh \| 100.00 \| 0 \| 0.01 \| \| Lakshadweep \| 100.00 \| 0 \| 0.003 \| | \|  \| Cumulative \| Births \| Percentage \| \| --- \| --- \| --- \| --- \| \|  \| percentage \| ('000) \| distribution \| \|  \|  \|  \|  \| \| India \|  \| 2,488 \| 100.00 \| \| Uttar Pradesh \| 18.59 \| 462 \| 18.59 \| \| Bihar \| 31.37 \| 318 \| 12.78 \| \| Maharashtra \| 41.87 \| 261 \| 10.50 \| \| West Bengal \| 50.24 \| 208 \| 8.37 \| \| Madhya Pradesh \| 57.28 \| 175 \| 7.04 \| \| Rajasthan \| 62.70 \| 135 \| 5.43 \| \| Gujarat \| 67.60 \| 122 \| 4.90 \| \| Tamil Nadu \| 72.15 \| 113 \| 4.55 \| \| Odisha \| 76.40 \| 106 \| 4.25 \| \| Andhra Pradesh \| 79.14 \| 68 \| 2.74 \| \| Karnataka \| 81.68 \| 63 \| 2.54 \| \| Chhattisgarh \| 84.11 \| 61 \| 2.43 \| \| Assam \| 86.53 \| 60 \| 2.41 \| \| Jharkhand \| 88.49 \| 49 \| 1.96 \| \| Punjab \| 90.16 \| 42 \| 1.68 \| \| Haryana \| 91.83 \| 41 \| 1.67 \| \| Telangana \| 93.47 \| 41 \| 1.64 \| \| Nct of Delhi \| 95.09 \| 40 \| 1.62 \| \| Kerala \| 96.42 \| 33 \| 1.33 \| \| Uttarakhand \| 97.30 \| 22 \| 0.88 \| \| Himachal Pradesh \| 97.84 \| 13 \| 0.54 \| \| Tripura \| 98.31 \| 12 \| 0.47 \| \| Jammu & Kashmir \| 98.71 \| 10 \| 0.40 \| \| Meghalaya \| 99.04 \| 8 \| 0.34 \| \| Manipur \| 99.23 \| 5 \| 0.18 \| \| Goa \| 99.30 \| 2 \| 0.07 \| \| Puducherry \| 99.36 \| 1 \| 0.05 \| \| Chandigarh \| 99.41 \| 1 \| 0.05 \| \| Dadra & Nagar Haveli & Daman & Diu \| 99.45 \| 1 \| 0.04 \| \| Arunachal Pradesh \| 99.49 \| 1 \| 0.04 \| \| Mizoram \| 99.52 \| 1 \| 0.04 \| \| Nagaland \| 99.56 \| 1 \| 0.04 \| \| Sikkim \| 99.59 \| 1 \| 0.03 \| \| Andaman & Nicobar Islands \| 99.60 \| 0 \| 0.02 \| \| Ladakh \| 99.61 \| 0 \| 0.01 \| \| Lakshadweep \| 99.61 \| 0 \| 0.001 \| |

## Table S11: Sample distribution of low birth weight and small size at birth and prevalence across demographic and socioeconomic characteristics, 2021, India

|  | Low birth weight | | | | Small at birth | | | |
| --- | --- | --- | --- | --- | --- | --- | --- | --- |
|  | Sample |  | Prevalence |  | Sample |  | Prevalence |  |
|  | distribution | % | % | 95% CI | distribution | % | % | 95% CI |
|  |  |  |  |  |  |  |  |  |
| Child sex: |  |  |  |  |  |  |  |  |
| Male | 108,713 | 52.10 | 17.03 | 16.69, 17.38 | 118,856 | 51.96 | 10.16 | 9.86, 10.47 |
| Female | 100,553 | 47.90 | 19.56 | 19.19, 19.92 | 110,519 | 48.04 | 11.39 | 11.06, 11.73 |
| Place of residence: |  |  |  |  |  |  |  |  |
| Urban | 44,381 | 27.66 | 17.36 | 16.80, 17.94 | 46,798 | 26.75 | 10.43 | 9.88, 11.01 |
| Rural | 164,885 | 72.34 | 18.58 | 18.29, 18.87 | 182,577 | 73.25 | 10.87 | 10.58, 11.17 |
| Social caste: |  |  |  |  |  |  |  |  |
| Schedule Caste | 42,754 | 22.82 | 19.54 | 18.99, 20.09 | 47,173 | 23.24 | 11.40 | 10.88, 11.94 |
| Schedule Tribe | 40,161 | 9.92 | 18.76 | 18.03, 19.51 | 45,733 | 9.97 | 10.41 | 9.80, 11.05 |
| Other backward classes | 81,215 | 43.45 | 17.79 | 17.40, 18.18 | 88,130 | 43.53 | 10.25 | 9.90, 10.60 |
| None of Them | 33,966 | 18.29 | 17.32 | 16.69, 17.96 | 36,234 | 17.85 | 11.23 | 10.62, 11.87 |
| Don't Know | 1,340 | 0.88 | 25.13 | 21.83, 28.75 | 1,568 | 0.93 | 12.51 | 10.28, 15.15 |
| Missing | 9,830 | 4.64 | 17.35 | 16.11, 18.68 | 10,537 | 4.48 | 10.76 | 9.61, 12.01 |
| Mother's level of education: |  |  |  |  |  |  |  |  |
| No Education | 40,933 | 18.61 | 20.10 | 19.55, 20.66 | 49,629 | 21.03 | 11.27 | 10.77, 11.78 |
| Primary | 25,890 | 11.88 | 20.63 | 19.88, 21.40 | 29,419 | 12.26 | 11.92 | 11.30, 12.56 |
| Secondary | 111,533 | 52.69 | 18.24 | 17.89, 18.60 | 118,661 | 50.93 | 10.88 | 10.55, 11.22 |
| Higher | 30,910 | 16.82 | 14.50 | 13.94, 15.08 | 31,666 | 15.78 | 8.74 | 8.24, 9.27 |
| Household wealth quintile: |  |  |  |  |  |  |  |  |
| Poorest | 51,248 | 22.07 | 20.92 | 20.39, 21.46 | 61,484 | 24.27 | 12.16 | 11.67, 12.67 |
| Poorer | 48,569 | 21.52 | 19.55 | 19.04, 20.07 | 53,579 | 21.71 | 11.60 | 11.12, 12.08 |
| Middle | 42,147 | 20.18 | 17.47 | 16.94, 18.02 | 44,692 | 19.63 | 10.46 | 10.00, 10.95 |
| Richer | 37,376 | 19.42 | 17.04 | 16.41, 17.68 | 38,858 | 18.53 | 9.84 | 9.31, 10.38 |
| Richest | 29,926 | 16.81 | 15.36 | 14.72, 16.03 | 30,762 | 15.86 | 8.86 | 8.33, 9.43 |

## Figure S7. Relationship between headcount and prevalence (%) of low birth weight and small size at birth, 2021

Notes: State labels were displaced by at most 10% on each axis for readability. Andaman & Nicobar Islands (AN), Andhra Pradesh (AP), Arunachal Pradesh (AR), Assam (AS), Bihar (BR), Chandigarh (CH), Chhattisgarh (CG), Dadra & Nagar Haveli & Daman & Diu (DH), Goa (GA), Gujarat (GJ), Haryana (HR), Himachal Pradesh (HP), Jammu & Kashmir (JK), Jharkhand (JH), Karnataka (KA), Kerala (KL), Ladakh (LK), Lakshadweep (LD), Madhya Pradesh (MP), Maharashtra (MH), Manipur (MN), Meghalaya (ML), Mizoram (MZ), Nagaland (NL), Nct of Delhi (DL), Odisha (OR), Puducherry (PY), Punjab (PB), Rajasthan (RJ), Sikkim (SK), Tamil Nadu (TN), Telangana (TL), Tripura (TR), Uttar Pradesh (UP), Uttarakhand (UK), West Bengal (WB).
